# Terminological Resources for Biologically Inspired Design and Biomimetics: Evaluation of the Potential for Ontology Reuse

**DOI:** 10.3390/biomimetics10010039

**Published:** 2025-01-09

**Authors:** Dilek Yargan, Ludger Jansen

**Affiliations:** 1 Institute of Philosophy, University of Rostock, 18051 Rostock, Germany; ludger.jansen@uni-rostock.de; 2 PTH Brixen College, Piazza Seminario 4, 39042 Bressanone, Italy

**Keywords:** computer-aided biomimetics, knowledge representation, ontology, taxonomy, thesaurus, function

## Abstract

Biomimetics aims to learn from living systems to develop innovative technical artefacts. As it transcends disciplinary boundaries and needs to integrate both biological and technological knowledge, a domain ontology for biomimetics would be highly desirable. So far, several terminological resources have been designed to support the biomimetic development process. This paper examines nine resources for Biologically Inspired Design and biomimetics, including taxonomies, thesauri, and ontologies. Their benefits and limitations for structuring or organising biomimetic knowledge are evaluated against nine criteria, including availability, clarity, and machine readability. Our analysis shows that existing terminological resources have little to no potential for reuse due to inconsistent structure, ambiguous class labels, lack of standardisation, and lack of availability. Furthermore, no resource adequately represents biomimetic knowledge, as all resources suffer from limitations in content representation, reusability, or infrastructure. In particular, an adequate domain ontology for supporting biomimetic development is lacking; we discuss the desiderata for such an ontology.

## 1. Introduction

Biologically Inspired Design (BID) is the attempt to learn from living systems for technical solutions. Bio-inspiration comes in a wide range of varieties, among which are biomimicry and biomimetics. Terminology is not always strict, but, in official guidelines, biomimetics has been defined as the endeavour to find innovative engineering solutions “through the abstraction, transfer, and application of knowledge gained from biological models” [1]. In pursuing this goal, biomimetics transcends the disciplinary boundary between biology and engineering. To be successful, they have to integrate biological and technological knowledge. Peculiar challenges often arise from divergent disciplinary perspectives, methods, terminology, and reasoning in biology and engineering, respectively. For instance, a transfer from living systems to technology is not trivial. Biology thrives on descriptive, context-sensitive language to capture the complexity of living systems, encompassing their physiology, morphology, ecology, and behaviour. In contrast, technology thrives on prescriptions and relies on standardised terminology to enable replicable design and clear communication. Translating the multifaceted descriptions of biological systems into actionable insights for technological applications requires an understanding of the distinct organisational structures of the two domains. As a result, the translation process involves not only bridging linguistic differences but also comes with the challenge of integrating diverse data sources from both domains for knowledge representation and eliciting.

In the last decades, ontologies have been suggested as a “silver bullet” [2] for securing the integration and interoperability of diverse data sets. Biology, like so many other fields, has, to a wide extent, transformed into a data-centred research discipline [3]. To secure solid data semantics and interoperability of diverse sets of data, ontologies are now widely used in data-intensive fields like the life sciences and medicine [4]. The Open Biological and Biomedical Ontology (OBO) Foundry (https://obofoundry.org, 25 December 2024) provides a framework for collecting and collaboratively developing orthogonal reference ontologies for these purposes [5]. A lighthouse project in this context is the expert-curated Gene Ontology, without which genetic research would hardly be possible today [6]. A comparable suite of ontologies is under development for manufacturing and engineering industry needs under the auspices of the Industrial Ontologies Foundry (https://www.industrialontologies.org, accessed on 3 January 2025). Therefore, it would be desirable to have an ontology to support the biomimetic development process. Such an ontology would promote the interoperability of diverse datasets from biology and technology, fostering the cross-disciplinary collaboration essential for biomimetics.

Up to now, various creativity methods and knowledge representation tools have been used to enhance biologically inspired or biomimetic development processes. Lists of such tools can be found in the review in [7] and in table 1 of [8]. Wanieck et al. [9] analysed in which steps of the biomimetic development process these resources can be used. In this paper, we will restrict our analysis to the terminological resources and evaluate their potential for being used as an ontology integrating biomimetic databases, or for being reused or re-engineered as such an ontology, or as a part of it. In order to do so, we first briefly characterise the differences between a taxonomy, a thesaurus, and an ontology as different kinds of terminological resources (Section 2). We then briefly discuss our methodology, including descriptions of our evaluation criteria (Section 3). We then evaluate biomimetic taxonomies and thesauri and ontologies in due course, either as used as schemas or developed explicitly for biomimetics (Section 4). We summarily discuss the results of our evaluation (Section 5) and conclude by pointing out possible routes for future research (Section 6).

In the following, labels for relations are in bold and lowercase (e.g., **exemplifies** or **has part of**), while labels for classes are written in italics (e.g., *encode* or *BMO*:*data*). In all other respects, we follow the resources discussed, while our default will be to use lowercase for the initials of class labels. We use colons to divide namespaces (like “*BMO*”) from class terms (like “*data*”). Sometimes, we use “<” to symbolise the **is a** relation, and “>” for its inverse, the **subsumes** relation, i.e., “*A* < *B*” means that the class *A* is subsumed by the class *B*, i.e., any instance of *A* is also an instance of *B*. “*A* > *B*” means that the class *A* subsumes the class *B*; in other words, any instance of *B* is an instance of *A*.

## 2. Terminological Resources: Taxonomy, Thesaurus, and Ontology

There are different kinds of terminological resources. Standardly, taxonomies, thesauri, and ontologies are distinguished. These three distinct yet related types of terminological resources offer different structures and methodologies for understanding and categorising data within a particular domain. Depending on their kind, they are encoded in human- or machine-readable formats and can be used as knowledge-structuring tools for organising, managing, and retrieving information to facilitate knowledge representation, semantic interoperability, and automated inferencing. Not all tools are fit for all purposes, though. Adapting McGuinness’s “ontology spectrum” [10], domain knowledge representation begins with creating a *controlled vocabulary* or a glossary, i.e., lists of terms with or without their meanings. These resources should provide unambiguous semantic interpretations for the terms used to describe a certain domain. From here, several steps can be taken to add additional semantical and logical information and thereby to increase the expressiveness of the domain representation. Each successive step results in a different terminological resource.

The first step is to transform the list of terms into an explicit hierarchy of narrower and broader terms. More specifically, a *taxonomy* is created to classify the entities of a domain monohierarchically with the **is a** relation. It offers a systematic way of organising domain entities, subsuming them under more expansive categories. In information systems, particularly, a taxonomy facilitates navigating large datasets and finding relevant information for, to name some, text classification and qualitative content analysis [11]. Taxonomies allow for a minimum of reasoning and the inheritance of properties from kinds to subkinds.

**Figure 1:**
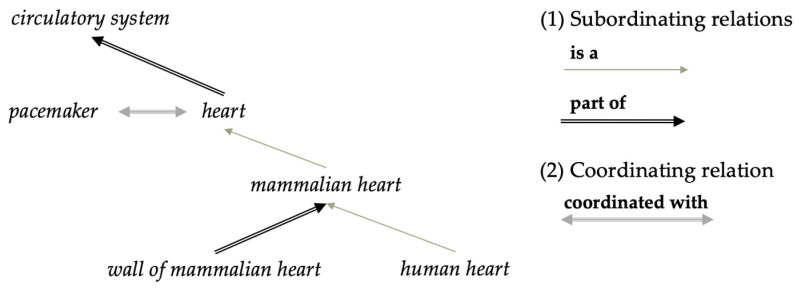
Illustration of the two types of relations in a thesaurus: (1) asymmetric and transitive subordinating (**is a** and **part of**) and (2) symmetric and associative coordinating (**coordinated with**) relations.

A *thesaurus* provides a controlled vocabulary of domain-specific terms and their synonymous, antonymous, meronymous, and related words with, but not limited to, equivalence, hierarchical, and associative relations between them [12,13]. Going beyond a simple taxonomy, a thesaurus can be structured poly-hierarchically. Thesauri standardly know two kinds of relations: an asymmetric and transitive subordinating relation (fusing both the subsumption relation and the part-of relation) and a symmetric associative relation (Figure 1), thus drastically increasing expressibility and reasoning power [14]. Thesauri are typically used, for example, for literature searches where they can support search extensions or suggest more restricted search terms. They are also used for various machine learning tasks, such as automatic indexing, sentiment classification, and coordination between datasets and models [15,16].

A shortcoming of thesauri is that they cannot distinguish between subkinds and parts. Obviously, this is an important distinction: *human heart* is a kind of *mammalian heart*, while *wall of mammalian heart* is not. *Ontologies* overcome this problem through explicit logical characterisations of relational properties with a specific semantic. In particular, ontologies will standardly distinguish between the **is a** relation and the **part of** relation. An ontology represents domain-specific entities, their interrelations, and axioms and definitions of entities and relations. In total capacity, it can support automated inferences and knowledge discovery through its rich and formalised representation of knowledge. In virtue of this feature, ontologies can serve as a *lingua franca* in information systems by providing a standardised framework for communication and information exchange among different groups, organisations, and alike.

Another family of terminological resources comprises schemas and data models. These share important features with ontologies but normally are much smaller. A *data model* defines entities, relations, and rules within a domain, acting as a blueprint for storing and accessing specific data [17]. A *schema* specifies entities, relations, and rules with greater detail and represents metadata hierarchically. Data models and schemata are used to specify the semantics of certain data sets and to allow certain inferences, but they are not intended to model the terminology of a whole domain.

## 3. Methods

### 3.1. Identification of Resources

In order to identify terminological resources for biomimetics, we carried out a literature review using Google Scholar. As search terms, we used “biomimetics”, “bioinspired design”, “bio-inspired design”, “biologically inspired design”, “biomimicry”, and “bionics” in combination with the terminological resource terms, namely “taxonomy”, “thesaurus”, and “ontology”. The searches were conducted in iterated rounds from August 2023 to August 2024. We also checked [8,9], which contain lists of tools and resources created for biomimetics.

We excluded any terminological resource that might be used in the biomimetic process but was initially built for other purposes in biology or technology. We found nine resources: one taxonomy, one thesaurus, two schemata, and five ontologies of various sizes (see Table 1). Determining the type of resources is not always trivial, as terminology varies (“schema”, “model”), and terms like “taxonomy” or “ontology” are sometimes used in a quite liberal way. Most of the biomimetic terminological resources discussed in this work were created for human comprehension, enabling humans to draw inspiration from biology. We examine all resources in terms of their potential to be reused or re-engineered, as their know-how is better used in future work. We thus did not exclude obsolete resources, as it might still be possible to learn something from them for the construction of a domain ontology for biomimetics.

### 3.2. Evaluation Criteria

In order to evaluate these resources, we oriented ourselves to existing lists of evaluation criteria. Several such lists of evaluation criteria and standards for terminological resources can be found in the literature. A challenge in our context is the diverse nature of the resources. We used the following to establish our criteria for evaluating biomimetic terminological resources:

- For all kinds in general: [18,19,20].
- For taxonomies: [11,21,22].
- For thesauri: [12,13,14,15,23].
- For ontologies: [5,24,25,26,27,28,29,30] and the good practice principles of the Open Biological and Biomedical Ontology Foundry (https://obofoundry.org, accessed on 3 January 2025).

From these, we first excluded all criteria requiring quantitative measurements that are difficult to operationalise, such as generality and appropriateness. Secondly, criteria such as relevance and novelty, which require the existence of other tools for comparison, are omitted due to the limited number of biomimetic tools available. Thirdly, criteria that require domain expertise both in technology and biology, such as maintenance and adequacy, are not included in the list. Lastly, criteria such as reliability and usefulness are also excluded because their evaluation requires an examination of application results.

Some of our criteria are not standardly required for some types of terminological resources. For instance, taxonomy inclusion is not always necessary in thesaurus design; machine readability and interoperability are not standard criteria for evaluating either taxonomies or thesauri; and documentation for the usability of taxonomies is typically not required, as their structure is often considered trivial. As we want to evaluate the potential for reuse in the context of an ontology, we nevertheless check these criteria for all types.

Table 2 presents a comprehensive overview of the criteria, along with their descriptions and measurement methods, used for evaluating biomimetic terminological resources. We grouped our criteria into three categories: infrastructure, content representation, and reusability.

**Infrastructure** criteria check whether the material is accessible for use and evaluation. **Availability** ensures that the resource is accessible or usable when needed. **Documentation** checks whether the resource is well-documented by providing precise and comprehensive instructions for supporting users, facilitating understanding and proper utilisation for both domain experts and non-experts. Documentation is evaluated by investigating any means of explaining the resource, such as GitHub repositories, webpages, or research papers.

**Content representation** comprises criteria that evaluate whether the formal, syntactic structure and content representation of ontologies, taxonomies, and thesauri are precise and correct. Among these, **taxonomy inclusion** checks whether there is the **is a** hierarchy culminating in mutually exclusive top-level terms and whether it is semantically correct. As sciences are becoming increasingly data-driven, the **is a** hierarchy becomes more and more important for (1) enhancing systematic literature searches or knowledge retrieval by offering narrower or broader search terms, helping navigate complex domains such as biology, (2) facilitating interoperability through alignment of different vocabularies, and (3) ensuring the semantic precision and accuracy by delineating term definitions through the taxonomy. This criterion can only be evaluated through semantic analysis, which can be conducted manually. Put formally, starting from the top-level terms, say *n_i_*, for each following node, *n_i+_*_1_, it is checked whether *n_i+_*_1_ **is a** *n_i_*, where *n*_1_ is the highest genus, and 1 ≤ *i* ≤ *m*, with *m* being the length of the longest branch. When all the nodes, except the top-level ones, of the terminological resource comply with this condition, then the resource passes this criterion.

**Logical consistency** verifies that the terminological resource is internally consistent and does not contain conflicting labels, statements, or definitions. This criterion is critical as conflicts or inconsistencies impede using and reusing a terminological resource. The assessment of this criterion is tool-dependent. For instance, we use automated reasoning to access this criterion with machine-readable resources.

**Consistent labelling** checks the adherence to coherent conventions for labelling entities and relations, as rules for labelling ensure faster term recognition and string matching. Unclear labels can impair readability and discovery in the hierarchies and hinder alignment and integration with other systems (cf. [26] for ontologies and [15] for thesauri). We check consistent labelling in two steps. First, we check internal consistency by manually checking whether labels are explicit, unambiguous, and context-independent (as far as possible) and whether homonymous terms and conjunctions in the name formulation are avoided. Consistent labelling also includes using a uniform format to separate words (e.g., white spaces, hyphens, or camel case), applying either singular or plural nominal forms (e.g., either *Animals* > *Cats* or *Animal* > *Cat*), using either lower- or uppercase (e.g., either *Animal* > *Cat* or *animal* > *cat*), and distinguishing classes from relations (e.g., by using **bold** for relations and *italics* for classes, or camel case for relations and kebab case for class labels). Second, we check adherence to the naming conventions of the OBO Foundry in particular [26], which serve as a gold standard to enhance the reusability of terminological resources. According to these recommendations, entity labels should be short, memorable, and understandable, even for non-experts. The labels must be univocal, avoiding homonyms to prevent ambiguity. Additionally, conjunctions in the label formulation should be avoided, and labels should be expressed in a positive form. Catch-all terms are discouraged in favour of specific labels that adhere to the genus–differentia labelling style. Using spaces as word separators is recommended, along with fully expanding abbreviations, acronyms, and special symbols into words. The guideline also advises against character formatting, prefers lowercase beginnings, and recommends the use of singular noun forms for label consistency.

**Clarity** goes beyond consistent labelling in evaluating how clearly and effectively the terminological resource communicates the intended meaning of the terms. Human understandability is of utmost importance for the use of terminological resources by human curators or annotators. The evaluation of this criterion is twofold. First, we investigate whether terms are unambiguous and easily understandable and whether they consistently convey the same meaning in every instance of use and refer to the same categories of entities in the real world. Second, if provided or extractable, we manually check definitions of terms, relations, and annotations.

**Reusability** criteria, finally, check features relevant to the reusability of the terminological resource as (part of) an ontology for biomimetics. First of all, **machine-readability** ensures that the terminological resource is represented in a formal language that a computer can process to apply, for example, automated reasoning and other computational processes to the content. Many of these formats are variants of mark-up languages. One such example is the Resource Description Framework (RDF) (https://www.w3.org/RDF, accessed on 25 December 2024). For ontologies, a standard format is currently the Web Ontology Language (OWL) [31]. When this criterion can be assessed through the documentation of the resource only, we cannot evaluate the tool in depth.

**Interoperability** evaluates how well a terminological resource is prepared to be used not only together with other semantic tools—including other terminological resources—but also in varying models and protocols to exchange data within and across domains [20]. Without delving into details of interoperability types and corresponding measurement models and standards (cf. [18]), we focus on the semantic and syntactic interoperability of the biomimetic terminological resources. To this end, we manually evaluate the internal consistency of these resources and their ability to facilitate information sharing with other semantic tools, including machine learning systems. This involves checking (i) whether the resource is created using existing standards (e.g., Dublin Core, SKOS, RDF, ISO 25964, or the OBO Foundry Principles) and (ii) whether the ontology, in particular, is built upon a top-level ontology.

**Extensibility** assesses the ability of a terminological resource to add or remove data sources with minimal effort [19]. This also includes the capacity to add or remove new terms or relations without needing to modify the taxonomy and/or axioms. We evaluate (i) whether guidelines or best practices are provided for making extensions and (ii) whether the terminological resource is built in a modular way.

While we aimed at independent criteria, it was not possible to avoid some overlap and interrelatedness between the criteria. For instance, the assessment of availability or machine-readability is straightforward; no other criterion affects their evaluation or is affected by their evaluation. Logical consistency, on the other hand, does not require checking the taxonomy inclusion, yet an inconsistent taxonomy implies logical inconsistency. Also, ambiguous and inconsistent term representations hinder interoperability, and depending on the complexity of the terminological resource, thorough documentation is crucial for reuse.

## 4. Evaluation Results

With the criteria and their fulfilment procedure set, we now describe the results of the evaluation of the terminological tools for biomimetics.

### 4.1. The Biomimicry Taxonomy

The Biomimicry Taxonomy has been developed by the Biomimicry Institute to organise the biological content of the website AskNature (www.asknature.org, 25 December 2024) [32]. Serving as “a source of inspiration for biomimetic design”, AskNature offers a publicly accessible database containing over 1750 biological strategies—the solutions to challenges in nature—across various levels of granularity, mainly sourced from peer-reviewed journals [32,33,34] and more than 300 technologies mimicking nature (www.asknature.org, accessed on 25 December 2024). Serving as the backbone of the AskNature database, the Biomimicry Taxonomy is developed by and for experts in the Biomimicry Institute to structure the biological data by functions, but it is often also recommended (and used) as a creativity tool to discover relevant strategies in the AskNature database.

The Biomimicry Taxonomy resulted from a huge amount of human labour: carefully chosen biological data are examined for function patterns and organised according to functions. It is a three-level hierarchical classification. Biological functions are classified into the eight highest genera, or “groups”, of functions: *Move or stay put*, *Maintain physical integrity*, *Maintain community*, *Modify*, *Make*, *Process information*, *Break down*, and *Get*, *store*, or *distribute resources*. These function groups are further divided into 30 subgroups, and these subgroups are then further divided, resulting in 162 functions, representing the final functional terms according to which biological information is categorised. According to this taxonomy, for example, the biological strategy of birds having dorsal ventricular ridge regions with neuronal connectivity similar to that in the neocortex of mammals addresses the functional challenge, which is classified (using two of the three levels only) as *Process information* > *Compute*. The function of finding nourishment fulfilled by brainless slime moulds by creating smart networks can be classified as *Perform* > *Process signals* > *Respond to signals* (see Table 3). The function terms given in the outmost right column of Table 3 are not themselves part of the three-level hierarchy but labels for the respective slots, which are used by the AskNature curators to categorise the biological strategies and can thus be used by the biomimetic researchers to navigate through the database. The list is not explicitly documented; for this reason, we harvested it by crawling the webpage. The function level is expected to express function terms nested under the subgroup level. Sometimes, the function level has an empty slot, i.e., the subgroup is a leaf node, the function term is the leaf node (see, e.g., the first row of Table 3). The leaf node of the function level does not necessarily provide the function label; the corresponding function term can be the combination of the leaf node and its parent node (the second and the third rows of Table 3) or the leaf node (see the fourth row of Table 3).

**Availability:** The Biomimicry Taxonomy is available in human-readable format at https://asknature.org/resource/biomimicry-taxonomy (accessed on 3 January 2025).

**Documentation:** The above webpage demonstrates how the taxonomy can be utilised in AskNature and in a biomimetic development process, and its methodology and some cases are documented by Vandevenne, Pieters, and Duflou [32]. However, neither the function terms nor their definitions are available through the taxonomy or explicitly documented. It is difficult to read the leaf nodes of the taxonomy, as one has to combine the levels to figure out the correspondent function term. Besides, the definitions of the function terms can be obtained through web crawling only; they are seemingly not created to be utilised by non-expert users. While the taxonomy is otherwise well documented, this is an obstacle to comprehending the taxonomy.

**Taxonomy inclusion:** In a taxonomy, the highest genera are mutually exclusive and represent types, and all nodes stemming from them should be subtypes. However, in this taxonomy, there are empty slots at the function level, and many labels at this level cannot be understood in isolation. They seem to be objects of verbal phrases (*Solids*, *Living materials*, *Time, and daylength*) or their adverbs (*Permanently*, *Temporarily*) or other attributes (*In*/*on solids*, *On demand*, *Over land*) rather than independent class labels. The supposed function label, then, can only be understood in combination with the other levels. For instance, the class label *Microbes* at the function level is not a function, but the function term *Protect from microbes*, which is itself not contained in the taxonomy, is a function a subtype of *Protect from living threats*. Additionally, in some cases, the function term simply repeats the term in the function level. If the labels are taken at face value, they obviously do not add up to the **is a** hierarchy (Table 3). In this respect, the Biomimicry Taxonomy deviates from being a conventional taxonomy.

**Logical consistency:** Due to the informal nature of the Biomimicry Taxonomy, logical consistency is difficult to evaluate. Under standard assumptions, however, the reoccurrence of one and the same class, such as *Gases* under different classes, would lead to an inconsistency within a taxonomy.

**Consistent labelling:** In light of the OBO Foundry Naming Conventions, the function labels of the Biomimicry Taxonomy are not only ambiguous but also not understandable in isolation (e.g., *In*/*on liquids*). The taxonomy uses plural labels (e.g., *Mineral crystals*) and does not adhere to the genus–differentia style. Additionally, several labels contain conjunctions (e.g., *Shape and pattern*), disjunctions (*Move or stay put*), slashes (e.g., *Size*/*shape*/*mass*/*volume*), or open enumerations (e.g., *Chemical* (*odor, taste,* etc.)), which should also be avoided.

**Clarity:** All three of *Chemical entities*, *Chemicals*, and *Chemicals* (*odor, taste,* etc.) are used without any noticeable difference in meaning; thus, it is not clear whether these terms have the same extensions or not. There are two pairs of terms, *Reproduce* (<*Make*) and *Self-replicate* (<*Reproduce < Make*), and *Physically assemble* (<*Make*) and *Physically assemble structure* (<*Physically assemble < Make*), which have the same definition. Additionally, the definitions of the terms do not follow a consistent pattern. For instance, the subtypes of *Chemically assemble* and *Chemically break down* are differentiated from each other via examples, which is the general approach of describing the terms in the Biomimicry Taxonomy; on the other hand, there are some terms defined with their general characteristics without a mention of their differentia from their sibling terms, such as *Compute* and *Coordinate*. Above all, the taxonomy falls short in terms of the readability of the leaf nodes, namely the function terms, which impairs human understandability. Consequently, readers are often left to make educated guesses.

**Machine readability:** The Biomimicry Taxonomy is not represented in a standardised machine-readable format.

**Interoperability:** Biomimicry Taxonomy fails to fulfil this criterion due to the lack of adherence to established taxonomy standards. Rather, the selection of biological strategies and the organisational structure of functions was subjectively based on the assessment of experts at the Biomimicry Institute [33].

**Extensibility:** The inflexible three-level structure of the Biomimicry Taxonomy and the hiddenness of its definitions are all obstacles when it comes to including possible extensions or including the taxonomy within a larger context.

### 4.2. The Engineering-to-Biology Thesaurus

Thesauri enhance information retrieval by offering alternative terms and suggesting broader or narrower concepts, improving the accuracy and comprehensiveness of search results. In biomimetics, thesauri often aim to facilitate communication by translating between biology and technology since the same terms are often used in biology and engineering with different meanings [35]. For instance, the term “clean” has different connotations in biology (e.g., “defend”) and engineering (e.g., “hygienic”) [36]; “structure” refers to the part of a building, bridge, or similar construction that supports and stabilises the entire form in structural engineering, but in biology, it refers to the underlying arrangement of parts that make up a specific morphological trait [37]; a term like “abscise” is biologically significant, but it is not easy to say to what it corresponds in the technical sphere [38]. Bridging these gaps between biology and technology requires expertise in both biology and technology. Biomimetic thesauri are intended to support developers in this effort.

In the literature, the “keywords” collected by Cheong et al. [39] and BiOPS (“Biology Inspired Problem Solving”) are sometimes considered thesauri [7,9] or as usable as such [39]. Cheong et al. [39] propose a retrieval algorithm to translate functional terms of the Functional Basis (see below) into biologically meaningful keywords. BiOPS is classified by Wanieck [40] as a technology–biology dictionary and by Weidner et al. [41] as a database that stores keywords from patents and websites and categorises the keywords in a technical and biological manner. However, neither has been developed explicitly as a biomimetic thesaurus. Moreover, neither the documentation nor the demo version of BiOPS has been available for inspection.

The Ontology-Enhanced Thesaurus (OET) is also not a thesaurus in itself but a suite of ontologies to facilitate the transfer of biological concepts into technology by integrating data across diverse fields with differing terminologies [42,43] (see Section 4.4.4 below).

The Engineering-to-Biology (E2B) Thesaurus is, then, the only “pure” biomimetic thesaurus to be evaluated. It is a Biologically Inspired Design tool that connects biological and engineering terms in a non-taxonomic hierarchical fashion [44,45]. The E2B Thesaurus consists of two parts: the collection of engineering terms in the Reconciled Functional Basis (RFB) and a collection of biological terms.

The RFB is the backbone of the E2B Thesaurus that serves as a framework through which engineering and biological terms are mapped. It combines the NIST Taxonomy with various versions of the Functional Basis developed by Little, Wood, and McAdams in 1997 [44]. The RFB follows the design paradigm of Pahl et al. [46], according to whom an artefact function is an intended input–output relation of a system whose purpose is to perform a certain task. Input and output are also called flows, i.e., a function can be described as the intended transformation of one flow into another. The RFB taxonomy is created as a standardised collection of taxonomies of flows and functions to encompass all the generic terms used in engineering design, according to which all the processes in a model are expressed in terms of functions and flows [47]. It is used in biomimetics because such a standardisation makes it easier to identify corresponding biological terms that can inspire innovative design solutions [48]. However, the E2B Thesaurus modifies RFB at certain points.

Table 4 illustrates the hierarchical structure of the E2B Thesaurus. It consists of two subthesauri, the E2B Thesaurus Function Terms and the E2B Thesaurus Flow Terms. The flow terms are, in turn, divided into Material Flow Terms, Energy Flow Terms, and Signal Flow Terms. All the thesauri organise the terms on three levels and align them to a collection of corresponding biological functions. The first level (called “Class”) consists of the highest classes of the taxonomy, and the second and third levels (called “Secondary” and “Tertiary”, respectively) provide more specific functions and definitions than the previous level [44]. *Branch*, *Channel*, *Connect*, *Control Magnitude*, *Convert*, *Provision*, *Signal*, and *Support* are the classes of the E2B Thesaurus Function Terms; *Material* is of the E2B Thesaurus Material Flow Terms, *Signal* is of the E2B Thesaurus Signal Flow Terms, and *Energy* is of the E2B Thesaurus Energy Flow Terms.

Secondary and tertiary terms are linked to a collection of biological terms, corresponding both to function terms and to flow terms. As biologically correspondent function terms, a list of verbs and process nouns were extracted and collocated with the RFB terms in a comprehensive biology textbook [44]. As biologically correspondent flow terms, nouns were selected from the textbook that were collocated at least twice with an RFB term; these terms were then sorted into signal, material or energy flow types [45]. The E2B Thesaurus uses italics to highlight biological terms if and only if the biological function or flow corresponds to multiple engineering functions or flows to highlight the relevance of these terms. Similarly, engineering terms are italicised if and only if they correspond to multiple biological terms [44].

**Availability:** The E2B Thesaurus is available in text form from various sources [44,48,49,50]. These versions differ in term inclusion, term labels, and the italicisation of the terms. For instance, in [48], there is no secondary term for *Human* under the Energy Flow Terms, whereas it is included in [44,49]. Additionally, the biological term for the tertiary term of *Optical* (<*Electromagnetic* < *Energy*) is *infrared radiation* in [48], where it is italicised. The same term occurs without italics [49], while in [44], it is simply “infrared” and not italicised. We analysed the thesaurus as documented on the developer’s webpage [51] as archived in [49,50].

**Documentation:** The construction details and the complete lists of the RFB taxonomies are available in [47], and the construction details and the E2B Thesaurus with an application example are available in [44,48,49,50]. The definitions of the terms are available in [47].

**Taxonomy inclusion** of the Reconciled Functional Basis has already been critically analysed by Garbacz [52], who notes that the taxonomy lacks guiding principles and thus fails to provide motivation for its primary and subsidiary divisions. The same problems hold true for the E2B Thesaurus. For instance, it is strange that *Convert* is both a class and a secondary term without any documented reason for this. Also, *Human* is neither *Energy* nor *Material*.

**Logical consistency:** At face value, this structure is logically consistent. There are problems, though. The non-exclusivity of certain function terms introduces overlaps in the E2B Thesaurus, which leads to ambiguity in the classification system. The non-exclusivity of subdivisions also introduces gaps in the comprehensive categorisation of functions. For instance, it is unclear how *Separate* differs from *Divide*; the latter seems redundant as it is defined as “to separate a flow” by Hirtz et al. [47]. Second, there is no specification of what distinguishes the secondary *Convert* from the parent class *Convert*. If there is at least one instance that is not an instance of the secondary *Convert*, then the first and the second *Convert* are not identical; parent and child classes should not share the same labels. Otherwise, the secondary class is redundant. Similarly, the class *Signal* in the Function Terms must be differentiated from the class *Signal* in the Signal Flow Terms.

**Consistent labelling:** The E2B Thesaurus does not comply with the OBO Foundry Naming Conventions. First, all the class, secondary, and tertiary terms begin with upper-case letters, but only the first entry of the Biological Function Correspondent Terms follows this convention. Second, *Allow DOF* is used, although “DOF” is not a widely known acronym. Lastly, only some of the correspondent Biological Function terms are nouns, such as “*DNA*”, “fragment”, or “recombination”, but most of them are verbs, which does not conform with the OBO Foundry Naming Conventions.

**Clarity:** The E2B taxonomy lacks guiding principles and thus fails to motivate its primary and subsidiary divisions, which introduces gaps in the comprehensive classification of the engineering terms. Inherited from RFB, this problem leads to ambiguity in the classification system [52]. The presence of unclear definitions and examples adds to the ambiguity. The subsumption relation in *Human* < *Material* and *Human* < *Energy* is also perplexing because a human is neither a material nor an energy. Also, given the sameness of class terms, it is not clear whether we deal with the same class here or with two different classes. What should be said here is probably that a human, either as a whole or in part, **participates in** the material flow or, in the case of the latter, that a human provides energy within the flow. Furthermore, the definition of the former denotes *Human* as “all or part of a person” whose biological correspondent is “body or being”. A part of a person can be their hands, which fulfil the function in an engineering design, but the biological correspondence does not reflect that. Moreover, “being” is a cover-all term, including all biological terms. Concerning the latter, the E2B Thesaurus does not suggest a corresponding biological flow term. Besides, *Human* is a specification of *Energy*, just as *Electrical* (see Table 4). The definitions intend to state “human energy” and “electrical energy”, respectively, but energy is not work but a capacity to do work. The definitions might be straightforward to an engineer, though they are descriptions that cause perplexity to non-experts. Additionally, it is not easy to figure out in what sense the biological terms correspond to multiple functions or flows. For instance, it may not be apparent to a researcher outside biological sciences in what sense “*DNA”*, like “*RNA”* and “*enzyme”*, **is a** *Particulate* and in what sense it **is a** *Composite*, where *Particulate* and *Composite* are supposed to be mutually exclusive categories under *Solid.* As a consequence, the E2B Thesaurus falls short in terms of this criterion.

**Machine readability:** The E2B Thesaurus is not machine-readable.

**Interoperability:** As the Reconciled Functional Basis provides common standards of functional representations, any semantic tool based on or utilised in the RFB taxonomy can be integrated into the E2B Thesaurus with ease. Moreover, there is a well-known example of the E2B Thesaurus being integrated with the BioMimetic Ontology (BMO; see Section 4.4.3 below), whose taxonomy is not based on the RFB: the Engineering to BioMimetic Ontology (E2BMO). The E2BMO is a biomimetic inference tool designed to facilitate user interaction with the BioMimetic Ontology (BMO), whose biological data are connected to biological function correspondents of the E2B Thesaurus through the Simple Knowledge Organisation System (SKOS) framework [53]. The successful integration of the E2B Thesaurus with an ontology (BMO), resulting in an inference tool (E2BMO), suggests that the E2B Thesaurus is interoperable with other tools. However, three main issues challenge this assumption.

The E2B Thesaurus does not adhere to usual thesaurus standards. Second, the biological correspondent terms of the thesaurus are not sufficiently structured, as the biological correspondent terms are not connected with each other, e.g., “RNA” should be said to be a hypernym of “mRNA” and “tRNA”. There is only a synonymy relationship between the secondary or tertiary terms of the RFB and the biological correspondent terms. As mentioned above, the inconsistent and unclear structure of the RFB taxonomy is inherited by the E2B Thesaurus. For instance, *Membrane* falls under the secondary classes *Solid–solid* (<*Mixture* < *Material*) and *Solid–liquid* (<*Mixture* < *Material*), which are mutually exclusive. Hence, the E2B Thesaurus cannot clarify how a particular membrane exemplifies a solid–solid or solid–liquid material in engineering terms. This inconsistency leads to errors when biological data are processed by an automatic reasoning program.

**Extensibility:** The E2B Thesaurus includes a standardised collection of flows and function taxonomies to encompass all the generic terms used in engineering design, which allows the integration of other biological thesauri constructed upon the RFB taxonomy. On the other hand, the unstructured nature of the biological correspondent terms hinders a consistent and clear integration.

### 4.3. Ontological Schemata for Biomimetics

Representing knowledge of interest in both human- and machine-readable formats, ontologies in biomimetics have been developed to expedite the exploration of biological functions and structures intended to correspond with technological counterparts and vice versa. This subsection reviews engineering design ontologies used in biomimetics without representing biomimetic knowledge itself.

#### 4.3.1. The Structure–Behavior–Function Schema (SBF)

The Structure–Behavior–Function (SBF) schema has been developed over the years to represent knowledge about a system’s structure, behaviour, and function [54]. It is based on Chandrasekaran’s [55] Functional Representation (FR) schema [56]. Terminology is not always strict; the SBF schema is also called the “SBF ontology”, the “SBF language”, or the “SBF model”. The SBF schema is said to serve as “a vocabulary of knowledge representation” or a knowledge representation language that provides an “ontology for teleological modeling” [57,58]. An SBF model thus represents the structure, behaviour, and functions of a biological or technical system via this “ontology” or “language” [56].

The SBF schema is employed in the Design by Analogy to Nature Engine (DANE) and Intelligent Biologically Inspired Design (IBID). DANE is an interactive knowledge-based design environment that supports Biologically Inspired Design [56,57]. The SBF schema provides a robust foundation for DANE’s organisational structure and functionality. DANE’s library contains SBF models representing biological or engineering systems [58]. IBID is a virtual librarian that helps designers of technological systems find relevant biological literature related to their design problems [59]. IBID uses the SBF schema to extract information about biological systems’ structure, behaviour, and function from their natural language descriptions and annotates biology articles with the SBF annotations, which are then used to retrieve relevant biology articles for design queries [60].

SBF consists of three main components: structure, behaviour, and function [56]. All three consist of corresponding subcomponents, with an abstract syntax for describing a system with SBF models and a list of rules and guidelines on interpretation [58]. The Structure Ontology, the basis of a Structure Model, consists of *Element*, whose subcategories are *Component*, *Substance*, and *Connection*. The Behavior Ontology, the basis of a Behavior Model, describes a system’s behaviour in terms of *States* and *Transitions*. Lastly, the Function Ontology, the basis of a Function Model, consists of *Primitive* and *NonPrimitive Functions*. All these subontologies and their components are connected to each other via certain relations, and there are certain ways of denoting the properties of the components.

**Availability:** The SBF schema is completely contained in [58].

**Documentation:** The SBF schema is documented in [58] on pages 25–29. For a model construction, see [58]; for an application, see [61]; and for an example, see [62]. There are, however, no elucidations for primitive terms for, e.g., functions and mechanisms.

**Taxonomy inclusion:** SBF contains an implicit taxonomy in so far as they list possible species names to talk about an instance of one of its general categories. For example, there is such a basic taxonomy for structures, with *Element* as the highest category and two mutually exclusive subcategories, *Component* and *Substance*. *Function* also has two subcategories, *Primitive* and *NonPrimitive*. However, *States* and *Transitions* are not proper subcategories of *Behavior* but are used to define behaviour, according to the idea that “*Behavior* is nothing more than a set of *States* and related *Transitions*” [58]: A system’s behaviour is expressed in terms of transitions between the states of an artefact. The Behavior Ontology does not have a proper taxonomy.

**Logical consistency:** The SBF schema appears logically consistent, as it contains grammatical restrictions only and no explicit axioms.

**Consistent labelling:** The SBF schema uses plural noun forms, and verb forms are used to label primitive functions. In light of the OBO Foundry Naming Conventions, several issues arise. Entity labels are unclear (e.g., it is not evident what distinguishes an instance of *Primitive Function* from an instance of *NonPrimitive Function*). *NonPrimitive* is a non-positive label, and both Pascal case (e.g., *CausalExplanation*) and camel case (e.g., *frictinallyEmbedded*) are used instead of separating words with white space. Uppercase letters also appear at the beginning of labels. Lastly, the genus-differentia labelling style is not reflected.

**Clarity:** The SBF schema is syntactically strictly formalised in the formal syntax of the Backus–Naur form. There are, however, neither semantic definitions of complex terms (e.g., *Behavior* or *Stimulus*) nor elucidations of primitive terms (e.g., *expel* or *ballAndSocket*) are given.

**Machine readability:** The formal syntax of the Backus–Naur form has been developed for use in computer applications like DANE. It should be possible to implement it in other formal languages.

**Interoperability:** The SBF schema can support interoperability, as it provides an integration framework. Various systems have been developed using the SBF models for design analysis and recommendations in DANE 2.0 [57]. Borgo et al. [63] showed that SBF can be connected with the top-level ontology DOLCE, and Jia et al. [64] created an information retrieval model called R-SBF based on the SBF schema.

**Extensibility:** The *Primitive Function* class comprises six function terms, whereas the *NonPrimitive Function* class is left to the user to populate. In several examples, the SBF schema has proved to be integrable with other ontologies. For instance, in IBID, mentioned above, not only the “subontologies”, e.g., the Function Ontology, can be modified and extended, but also additional “ontologies”, e.g., an environment ontology, can be included in the SBF schema. Besides, the UNified Ontology for BID (see Section 4.4.1 below) can be considered as an extension of both the SBF schema and the SAPPhiRE model.

#### 4.3.2. The SAPPhIRE Model

Rooted in the Function–Behavior–Structure (FBS) Ontology [7], the SAPPhIRE (State change–Action–Part– Phenomenon–Input–oRgan–Effect) model is a generic model to represent the causality of natural and artificial systems in order to explain the causality of natural and engineered systems by providing a structured framework for understanding the relationships between system components, behaviours, and environmental interactions [65]. For this reason, it is also called a causality model. The SAPPhIRE model is sometimes called an “ontology”, an “ontology-based representation”, or a “causal description language” [66,67,68].

Founded on the SAPPhIRE causality model, Idea-Inspire was developed as a computational tool for browsing a database of biological and artificial systems for inspiration and problem-solving in biomimetics. The latest version of the tool is the web-based version Idea-Inspire 4.0, whose representation system consists of a functional decomposition model, text, and digital support, along with the SAPPhIRE model [69].

As its acronym suggests, SAPPhIRE comprises seven disjoint categories, which cover the physical components of the system and inference, their interactions, and the scientific law that governs them. These categories are interrelated through defined relations; for instance, *action* **creates** *parts*; *organs* and *inputs* together **activate** *effects*, which **creates** *phenomena* [65]. 

**Availability:** The SAPPhIRE model is available in [65].

**Documentation:** The definitions of the main components of their model are documented in [65]. For a detailed explanation of the SAPPhIRE components and examples of model constructions, see [70], and for an application in product–service systems, see [71].

**Taxonomy inclusion:** The model does not specify any subclass relations, nor does it consider the usage of upper-level categories.

**Logical consistency:** SAPPhIRE is trivially consistent because it does not have any logical constraints to be checked.

**Consistent labelling:** Labels vary between lowercase (*organs*) and uppercase usage (*Physical effect*). Also, parentheses are used in labelling, e.g., (*change of*) *state* and (*current subsets of*) *parts*.

**Clarity:** Venkataraman and Chakrabarti [70] give definitions and clarifications of the classes of the SAPPhIRE model. However, some labels, like *Parts* or *Organ*, do not reflect everyday language and are difficult for non-experts to understand because the class definitions do not refer to real-world entities. For instance, *Parts* is defined as “the set of components and interfaces that constitute a system and its environment”, which is closer to a definition of a system design. The elucidation of *Phenomenon* is also problematic. First, it is defined as “an interaction between a system and its environment”, which is a deviation from standard use. Second, the displacement of a body is said to be an example of a *Phenomenon*, which is at most the result of such an interaction.

**Machine readability:** The model is human-readable but follows a formal structure that could be converted into a machine-readable format.

**Interoperability:** Due to its web of relations, the SAPPhIRE model provides a specific means for interoperability and has been used in various applications. However, it does not follow standard data formats or protocols.

**Extensibility:** The SAPPhIRE model has already been modified and extended over the years [72]. Moreover, the UNified Ontology for BID (see Section 4.4.1 below) can be considered as an extension of both the SAPPhiRE model and the SBF schema.

### 4.4. Ontologies Explicitly Built for Biomimetics

#### 4.4.1. The UNified Ontology for BID (UNO-BID)

The UNified Ontology for Causal–Function Modeling in Biologically Inspired Design, or the UNified Ontology for BID (UNO-BID), is a unified reference lexicon grounded in the ontologies of the function model of DANE, based on the SBF models, and the SAPPhIRE causal model (see Section 4.3 above). Rosa, Cascini, and Baldussu [72] propose the UNO-BID as a common semantic basis, embedding all the information from complementary and compatible approaches that enhance the compatibility, effectiveness, and flexibility of BID approaches.

The development of the UNO-BID began with a thorough analysis of DANE and the SAPPhIRE model to identify and harmonise shared and distinct terms and to form a hierarchy. The UNO-BID comprises two highest genera, after *Thing* of Protégé: *Abstract_Universe* and *Physical_Universe*. The former contains “all the things (subclasses) that are an ideal representation (model) of physical objects or abstract concepts, built based on these models”, while the latter contains “all the real things” (p. 201). *Abstract_Universe* has 18 subclasses, including *Function*, *Function_Abstraction*, *Cause*, *Action*, and *Causal*. Some of these subclasses have further subclasses, which are four in total. *Physical_Universe* has only three subclasses: *Environment*, *Interface*, and *System*. The latter two have further subclasses, which are four in total. 

Rosa, Cascini, and Baldussu [72] describe two relations between UNO-BID entities: **has_subclass** and **defined_by**. The former establishes the hierarchy; the latter indicates semantic dependencies between classes when defining the class labels. The **has_subclass** relation generates the taxonomy of the UNO-BID.

**Availability:** No data file has been available for inspection.

**Documentation:** The sole resource for comprehending and analysing the UNO-BID is [72]. Term definitions and their comparison with SAPPhIRE and SBF models are provided.

**Taxonomy inclusion:** The UNO-BID contains a class hierarchy structured by the **has_subclass** relation. However, the class hierarchy is not always adequate. For example, *Physical*_*Phenomenon* is defined as an “interaction between the system and its environment” but is not subsumed under *Interaction*. In contrast, *Causal* (defined as “A thing implying a cause”) should not be subsumed under *Abstract_Universe*. In fact*, Causal* is a modifier that has no place in an ontology at all. Note also that things do not imply anything, only propositions do; as defined, the class *Causal* is doomed to be empty.

**Logical consistency:** There is nothing that could create logical inconsistency.

**Consistent labelling:** Unlike the UNO-BID’s basis, namely, the SBF schema and the SAPPhIRE model, all term labels are in singular form, which can be observed in the graphical representation of the ontology (figures 9 and 10 of [72]). On the other hand, table 9 in [72] lists the UNO-BID definitions where some terms are not found in the graphical representation in the plural. Lastly, in light of the OBO Foundry Naming Conventions, whitespace should be used to separate words instead of *FinalState* or *Abstract_Universe*, and labels should begin with lowercase letters.

**Clarity:** The UNO-BID falls short in terms of clarity due to its ambiguous and imprecise class labels and definitions. For instance, *Element* is defined as an “atomic-level element of the system” ([72], p. 203), yet no information is provided on atomic and non-atomic level elements. *Causal* should not take place as a class, and it is odd that it shares the same level with *Cause*. Due to its definition, the above-mentioned *Physical*_*Phenomenon* not being a subclass of *Interaction* is also unusual. Similarly, *Universe* is defined as all “existing matter and space considered as a whole”, but *Abstract_Universe* is a *Universe* without encompassing matter and space. Lastly, there is a difference between the number of terms represented in figures 9 and 10 of [72] and table 9 of [72]. For instance, *Postcondition* and *Precondition* are types of *Condition,* but they are not nested under *Condition* in figure 9 of [72]; the definitions of *Initial_State* and *FinalState* [sic] are not given in table 9 of [72], and *Postcondition*, *Precondition*, and *State variables* are not included in figure 9 of [72].

**Machine readability:** Documented to be used together with the editor Protégé 4.3.0, the UNO-BID is machine-readable. Its native format is not specified, but once loaded into Protégé, it can be exported in various formats like OWL or RDF.

**Interoperability:** The UNO-BID appears to be interoperable with other semantic tools based on the SBF schema or the SAPPhIRE model, as well as with terminological resources that use its representation language in Protégé. However, unclear semantics, ambiguous term definitions, and an inconsistent taxonomy hinder its interoperability.

**Extensibility:** The UNO-BID has a modular design that could support extensibility.

#### 4.4.2. The Ontology for Bio-Inspired Design

Yim, Wilson, and Rosen [73] sketch the design pattern for an ontology for Biologically Inspired Design intending to capture, retrieve, and reuse bioinspired design solutions based on physical architectures, behaviours, functions, and strategies. Wilson et al. [74] present a more fully developed picture of this ontology that allows designers to store and retrieve biological and engineering solutions using this ontology. Although the authors did not explicitly name the ontology, we refer to it as “the Ontology for Bio-inspired Design” (Ontology for BID) based on the title of the paper.

The Ontology for BID consists of the six highest classes—*Flows*, *Actions*, *Attribute*, *System Strategy*, *Structure*, and *Domain*—with corresponding subtaxonomies. The “Flow (Driving Input and Functional Output) Taxonomy” includes modified flow terms from the Functional Basis (see Section 4.2 above) and is used to define the functions of the system. The Action Taxonomy includes modified functional terms from the Functional Basis and is used to define the behaviour of the system. The Attributes Taxonomy consists of properties that define the context for determining the states of the system. A study of mechanical engineering textbooks and reference books was conducted to categorise common properties to outline the Attributes Taxonomy. The System Strategy Taxonomy consists of defined classes that represent the means by which the behaviour of the system is performed. The Structure Taxonomy consists of classes representing the systems in which the strategy is performed. Finally, the Domain Taxonomy encompasses system domains, but biology and engineering are the only domains currently distinguished.

The Ontology for BID includes nine relations, such as **hasStructure**, connecting *System Strategy* to *Structure*, **hasStrategy**, connecting *Structure* to *System Strategy*, and **fromDomain**, connecting *System Strategy* to *Domain*. The **satisfiesFunction** relation has *System Strategy* as its domain and a function description as its range, defined through the **hasInput** and **hasOutput** relations. Similarly, the **refinesBehavior** relation has *System Strategy* as its domain and a behaviour description as its range, defined through the **hasAction** and **hasAttribute** relations.

**Availability:** Neither the entire ontology nor the machine-readable file is documented, the Structure Taxonomy is missing, and the other taxonomies are incomplete. Our evaluation is based on the limited information provided in the taxonomy samples, where the classes are organised monohierarchically, and in table 1 of [74], which lists the relations and their definitions.

**Documentation:** The ontology is not documented thoroughly. Details of a toy model are documented in [73], while fragments of taxonomies are only documented in [74].

**Figure 2:**
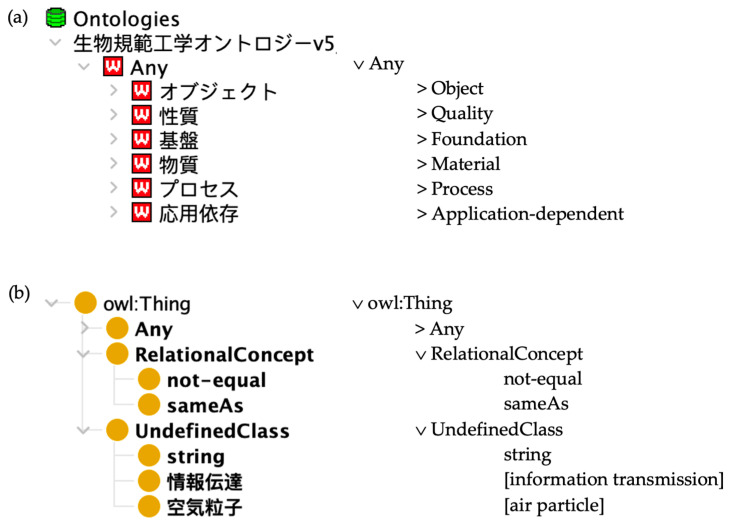
Top-level categories of the OET viewed in Hozo (**a**) and in the OLW export in Protégé (**b**), together with English translations on the right-hand side. Translations in brackets added by us.

**Taxonomy inclusion:** The Ontology for BID is designed to be a collection of taxonomies whose classes are interrelated with certain relations (see figure 4 in [74]). Only excerpts of the Flow, Action, Attributes, and System Strategy taxonomies are provided in figures 1, 2, 3, and 5 of [74], respectively.

As the Functional Basis is not strictly a taxonomy (see Section 4.2 above), neither the Flow Taxonomy nor the Action Taxonomy is considered a taxonomy in the strict sense for the same reasons. The Attributes Taxonomy has dubious connections, as the classes are not related to their superclasses by the **is a** relation. For instance, an instance of *Diameter* is not an instance of *Shape*, nor is an instance of *Pitch* an instance of *Angle*. Furthermore, in the entire taxonomy of the ontology, the classes are not mutually exclusive. *Mechanical* appears once in the Flow Taxonomy (*Mechanical* < *Energy* < *Flow*) and twice in the Attributes Taxonomy (*Mechanical* < *Extrinsic Properties* < *Attributes* and *Mechanical* < *Material Properties* < *Attributes*). Moreover, *Force* **is a** *Mechanical* both as an attribute and a flow. These instances lead to an inconsistent taxonomy structure. Thus, the Ontology for BID fails to satisfy taxonomy inclusion.

**Logical consistency:** The developers tested the logical consistency of the ontology with the RacerPro reasoner embedded in Protégé [74]. However, inconsistent taxonomy causes logical consistencies.

**Consistent labelling:** Although all the relation labels are chosen consistently, class labels vary in form. For instance, some labels for the highest classes are singular (e.g., *System Strategy*, *Structure*, and *Domain*), while others are plural (e.g., *Flows* and *Actions*). The same inconsistency appears in lower classes, such as *Shape* and *Material Properties*. Word separation methods are also inconsistent: dash (e.g., *Shape-Physical*), Pascal case (e.g., *PiezoElectric*), and Pascal case with underscore and dash (e.g., *Human_Musle-IsomContraction*) are used. The label *Position*/*Orientation* includes a “/”, which should be avoided.

Certain classes in the description logics (DL) formulations are different from their labels in the taxonomies. For instance, to differentiate the *Mechanical* class in the Flow Taxonomy, *Mechanical Energy* is written in the DL formulation. However, the corresponding taxonomy is not specified for the *Force* class in the DL formulations. In addition, word separation in the formulations varies between underscores (e.g., *Electrical_Energy*) and white spaces (e.g., *Mechanical Energy*). That said, labels in the ontology and formulations should match.

According to the OBO Foundry Naming Conventions, all class terms should be in lowercase and singular form; this convention is obviously not followed by the Ontology for BID. Additionally, the genus–differentia labelling style is inconsistently applied, particularly in the Attributes Taxonomy. Some class labels, particularly those of the System Strategy Taxonomy’s leaf nodes, are long and unintelligible to non-experts (e.g., *Human_Musle-IsomContraction*); such abbreviations should also be avoided.

**Clarity:** Although the Flow Taxonomy and the Action Taxonomy are familiar from the Functional Basis—with definitions of entities available in [47]—definitions for the entities in the remaining taxonomies are not provided. Limited information on the classes shows that *Force* appears in both the Flow Taxonomy and the Attributes Taxonomy, which suggests potential ambiguity or unobservable instances. While this could be an oversight, the **hasSystem** and **has_property** relations are defined in table 1 of [74] but referred to elsewhere (table 2 and figure 4 in [74]) by the labels **hasStructure** and **hasAttribute**, respectively. That said, table 1 in [74] includes relation labels that (i) are undefined (e.g., **has_function** and **has_behavior**) and (ii) vary in style (e.g., **satisfies_function** instead of **satisfiesFunction** and **has_input** instead of **hasInput**). Lastly, the abbreviations specific to this ontology should be avoided at all.

**Machine readability:** The ontology is machine-readable as it has been encoded in OWL using the Protégé editor.

**Interoperability:** The Ontology for BID is represented in a standardised language, namely OWL, which provides interoperability. Additionally, since the Functional Basis provides common standards of functional representations, any semantic tool based on or utilised in the Functional Basis can be integrated into the Ontology for BID. However, inconsistent taxonomy and ambiguity of the terms hinder full interoperability.

**Extensibility:** The modular nature of the ontology allows extensibility.

#### 4.4.3. The BioMimetic Ontology (BMO)

The BioMimetic Ontology (BMO) is a biomimetic tool that aims at systematically organising biological knowledge by employing the TRIZ method (named after the acronym for the Russian equivalent of “Theory of Solving Problems Inventively”) [75]. The TRIZ method addresses technical trade-offs by means of so-called inventive principles (IPs), which are creative solutions that have proven to help mitigate the trade-off in question in the past. According to Vincent [76,77], trade-offs are essential for detecting and comprehending biological adaptations; thus, trade-offs are fundamental not only to biology but also to technical problem-solving, which suggests the applicability of TRIZ in both fields. For this reason, the BMO tries to incorporate trade-off parameters and inventive principles for biomimetics [53,76,77,78]. The ontology is intended to facilitate identifying and aligning biological trade-offs and connecting engineering problems with biological solutions [79].

The BioMimetic Ontology integrates the Basic Formal Ontology (BFO) and has three highest genera: *BMO*:*data*, *BMO*:*trade-off,* and *BFO*:*entity*. The first two are reported to be used as repositories. The two most crucial categories of the TRIZ approach are categorised as follows: *BMO*:*definition of trade-off* < *BFO*:*specifically continuant dependent* and *BMO*:*inventive principles* < *BFO*:*process*. Their subcategories are chosen according to the mantra “things do things somewhere” [78], viz., “Do Things”, “Things”, and “Somewhere”. The BMO categories are rich in annotations, such as *BMO*:*inventive principles* are annotated by TRIZ numbers and examples of usage. As relations, the BMO uses some central BFO relations and imports other relations from the Relation Ontology, together with some custom-made relations; the most important ones are **parameter** and **applied the process**, which define *BMO*:*trade-off* entities and *BMO*:*data*.

Originally conceived as a system for solving technical problems and intended to provide universal solutions, TRIZ requires modifications when applied to biology, as biological contradictions do not always fit neatly into the classical TRIZ framework. The goal, therefore, was to integrate biological solutions into the TRIZ matrix, enabling engineers to retrieve answers without realising they were derived from biology. However, this approach has a cost: while retaining TRIZ terminology helps engineers maintain familiarity, it introduces ambiguity and taxonomical inconsistencies, as seen below.

**Availability:** The BioMimetic Ontology is only available upon request from J.F.V. Vincent, its principal developer, who shared the desktop version of the BMO and its formal description with us on 18 July 2023, and the WebProtégé version, the last visit to which was on 9 August 2024.

**Documentation:** The desktop version documentation is available upon request. The software also contains information about the ontology, yet the internal documentation is not complete.

**Taxonomy inclusion:** The BMO deviates in several respects from standard ontology development practices. As already mentioned, the two uppermost classes *BMO*:*data* and *BMO*:*trade-off* are asserted at the same level as *BFO*:*entity*, which is supposed to include all the other categories. Although they are reported to be used as repositories, these highest genera are not mutually exclusive. The *BFO*:*entity* branch falls short in terms of its taxonomic structure, as, e.g., *BMO*:*trustworthiness* should not be related to *BMO*:*wholeness* with **is a** relation, but its being a parameter about wholeness in a biological sense should be defined differently. A lot of subsumption relations in BMO are dubious. For example, *BMO*:*locomotion* is said to be a subclass of *BFO*:*disposition* instead of a *BFO*:*process*. *BMO*:*particle* and *BMO*:*fibre* are said to be *BFO*:*specifically dependent continuant* in the desktop version, and *BFO*:*generalically dependent continuant* in the WebProtégé version, while both should rather be below *BFO*:*independent continuant*. Thus, BMO lacks an adequate taxonomy.

**Logical consistency:** Consistency checks using the reasoner plugins HermiT 1.4.3.456 and Pellet within Protégé 5.6.3 yielded no logical inconsistencies within the BMO. We note, however, that the native BMO relations lack domain and range restrictions, which could impose additional challenges for the logical consistency of BMO.

**Consistent labelling:** Most of the time, inventive principles are inadequately labelled by verb phrases; noun phrases are used only sometimes, such as *local quality*, *functional reversal*, and *degree of specialisation*. Although most of the noun terms are singular, there are few plural terms, such as *inventive principles* and *plant defences*. Additionally, there is no consistent way of separating the words. To concretize the problem, see the examples of *under-fur*, *growth rate-lifespan trade-off*, *animal hormone*, and *introduce_hinges*. Some classes have somewhat unorthodox labels, like *ecdysial line of skin of a snake* (*Serpentes*). In light of the OBO Foundry Naming Conventions, *substitute a mechanical effect with something non-mechanical* is another problem case, as it is not only very long but also a verb phrase. Conjunctions and strange connectors are also used in the labelling, such as *perform a partial or excessive reaction*, *growth and stress*, *sexual* vs. *natural selection*. The genus–differentia labelling style is not preserved, such as it is not clear how *provide information for another organism* is different from its sibling classes of *animal behaviour*, *molecular behaviour*, and *plant behaviour*, all of which are subclasses of *behaviour*.

**Clarity:** Many class labels of the BMO are perplexing; this is often inherited from the TRIZ terminology. For example, *BMO*:*simple and easy process*, despite its label, is not *BFO*:*process*, but a subclass of *BMO*:*definition of a trade-off*, whose superclass is *BFO*:*continuant*, which is disjoint from *BFO*:*process*. Also, *BMO*: *lateral locomotory appendage part* and *BMO*: *lateral locomotory appendage* are both subclasses of *UBERON*:*appendage*, where Uberon stands for the Uber-anatomy Ontology [80]. The first entity is **part of** the second entity and is not a subclass of the third one. Other examples of the **is a** overload are *wall of mammalian heart* < *mammalian heart* < *heart* and *BTO*:*lymph* < *blood*, where BTO stands for the BRENDA Tissue Ontology [81].

A significant challenge is that domain and range constraints for the BMO-relations are insufficiently specified. For instance, *BMO*:*trade-off parameters* classified in *BFO*:*entity* defines some classes in *BMO*:*trade-off* with the relation **parameter**, whose domain and range are not defined, along with another BMO-specific relation, **apply the process**.

**Machine readability:** The BMO is machine-readable as it is represented in OWL both in desktop and web versions of Protégé. It can also be utilised in automated systems and computational processes; for instance, see its integration with the E2B thesaurus and resulting in the E2BMO in Section 4.2.

The **Interoperability** of BMO is facilitated by its import of BFO as a top-level ontology. However, its interoperability is seriously hampered due to its unconventional design decisions. Most prominently, it adds two highest genera, *BMO*:*data* and *BMO*:*trade-off,* outside of *BFO*:*entity*. Somewhat unorthodoxically, *BMO*:*data* serves as a list of references to the biological and biomimetic literature, while *BMO*:*trade-off* offers a small selection of TRIZ strategies. Also, the domain and range of its relationships are not defined properly, which hinders seamless interaction with other BFO ontologies.

**Extensibility:** The modular characteristics of the BMO facilitate its extensibility. As it imports BFO, BMO can be extended with other BFO-based ontologies.

#### 4.4.4. The Ontology-Enhanced Thesaurus (OET)

The Ontology-Enhanced Thesaurus (OET) has been developed to facilitate the transfer of biological models, systems, and elements to technology. It has been designed to integrate data from diverse domains, each with terminological differences [42,43]. The OET addresses this challenge by employing ontologies that allow for efficient semantic reasoning and integration with diverse biomimetic databases [43].

The OET can translate terms between domains by integrating any biomimetic thesauri. It is modularly composed of the Goal, Functional, Living Environment, Organism, and Property ontologies [42]. These ontologies play a crucial role in addressing the missing link problem that arises when two terms are semantically related but lack explicit connections, as they are designed to integrate various databases, allowing for information retrieval across diverse domains.

The OET is originally encoded in Hozo, an ontology format that can be displayed with the Hozo ontology editor (www.hozo.jp, accessed on 3 January 2025). To check reusability, we exported an OWL version of the OET and displayed it in Protégé 5.6.3. Class terms in the OET are primarily in Japanese, but the OET also provides labels in English, Italian, and French. Curiously, in one OWL export from the Hozo version, English labels are marked as “rdfs:label [language: it]”, while the Japanese labels were marked as “rdfs: label [language: en]”. When no English labels were available, we used Google and Yandex translators; when we use our own translations of terms, we display them in square brackets.

Surprisingly, the number of the highest classes varies according to the version used. In Hozo, the class *Any* subsumes six highest classes, while there are three highest classes in the OWL export, one of which is the class *Any*. Figure 2a shows the six top-level categories of the Hozo display together with their English translation: *Object*, *Quality*, *Foundation*, *Material*, *Process*, and *Application*-*dependent*, while Figure 2b shows the Protégé display of the OWL export with the additional classes *RelationalConcept* and *UndefinedClasses* together with their immediate subclasses, which are not to be seen in the Hozo version.

There is no clear alignment of these classes with the various ontologies described in the publications or the ISO/TR norm. Kozaki and Mizoguchi [42] describe two parts of their “biomimetics ontology”: the Functional Ontology (or the Ontology of Function) and a biological ontology that includes ontologies for Living Environment, Structure, Property, and Action. Kozaki and Mizoguchi [42] also mention that *Function*, *Goal*, *Behavior*, *Property*, *Structure*, *Organism*, and *Living Environment* are chosen as top-level categories. It is unclear why *Action* is excluded from the top-level categories, whether *Goal* belongs to the Functional Ontology, and why *Behavior* and *Organism* were not included in the biological ontology.

Later, ISO/TR 23845 introduces a new ontology called Functional Decomposition, along with the previously mentioned ontologies—Behavior, Living Environment, Functional, Structure, and Taxonomy of Creatures in figure 3 of [35]. However, figure 7 in [35] introduces the Species Ontology, which was not listed in figure 3 of [35]. Additionally, the Organism Ontology mentioned in previous work is also referred to as the Taxonomy of Creatures, and the Functional Ontology is subdivided into the Functional Concept Ontology and the Ways of Function Achievement Ontology, also called the Function Decomposition Ontology. To add to the complexity, Mizoguchi and Kozaki [43] distinguish between Functional Ontology and Function Decomposition Ontology, and they refer to *Property* as *Feature*.

The ontology file provided by the developers also makes it difficult to find out what the OET classes are exactly, as the descriptions in the publications and the ISO/TR document and the ontology files themselves are not easily aligned with each other. The highest classes of the file are not the mentioned top-level categories. For instance, *Living environment* is a subclass of the highest class *Space_region*, and the Organism Ontology is the *Creature* subbranch of the highest category *Object*. To investigate the OET ontologies, we searched all the subclasses of the highest categories in the OET file.

*Object* has four subclasses, one of which is *Organism*, whose subclass *Creature* is the uppermost class of the Organism Ontology (or the “Taxonomy of Creatures” or “Species Ontology”), which is used to align organisms with functions or structures [35]. *Creature* has three immediate subclasses: *Animal*, *Plant*, and *Bacteria*. *Quality* has three subclasses: *Structure*, *Characteristics*, and *Attribute*. The Structure Ontology is the *Structure* branch, which has twenty-eight immediate subclasses. What ISO/TR 23845 [35] calls the Property Ontology seems to be *Characteristics* branch, which has thirty-five subclasses. OET follows YAMATO in seeing properties as dependent entities other than relations [35,82]. *Foundation* has *time* [sic] and *Space_region* as subclasses. A subbranch of the latter is the Living Environment Ontology; it is a part of the OET because a particular environment can drastically affect organisms’ exercise of a particular function [43]. The upper-most class of the Living Environment Ontology, i.e., the class *Living_environment*, has five subclasses: *Aquatic*, *Brackish_water*, *Mud_lake*, *Pollen*, and *Terrestrial*. *Material* has three subclasses: *Light-receiving_material*, *Compound*, and *Three_states_of_matter,* and *Process* has three subclasses: *Phenomenon*, *Act*, and *Function*. The Function Ontology has nineteen classes, which include *Hiding*, *Control*, *Mechanical_function*, and *Information-related_functions. Application*-*dependent* has four subclasses: *Insect_mimetics*, *Output*_*Type*, *Expression,* and *Goal*. Finally, the Goal Ontology hangs down from its highest class *Goal*; it provides a hierarchy of *Functional_goal* and *Specific_goal*. The OET ontologies are interrelated by means of the relations **part-of** and **attribute-of**.

**Availability:** The OET ontologies are only available upon request from Riichiro Mizoguchi and Kouji Kozaki.

**Documentation:** The OET is documented in [35]. However, not much detail is provided about the ontologies involved, and it is unclear how the norm text matches the actual ontology file. The term definitions are not documented.

**Taxonomy inclusion:** For inspection of the OET ontologies, we used the Hozo and Protégé; see Figure 2. However, a more detailed analysis was difficult, as English labels are not provided for all classes. Nevertheless, it can easily be observed that the **is a** relation is not preserved throughout the taxonomy. For instance, an instance of *air particle* is not an instance of *UndefinedClass*, and an instance of *Output_type* is not an instance of *Application*-*dependent*.

**Logical consistency:** The reasoner Pellet displayed several errors and warnings and indicated that the exported OWL version of the OET is not logically consistent.

**Consistent labelling:** The class labels seem to be arbitrary. First, there is no consistent spacing, with variants such as hyphen (*Application-dependent*), underscore (*Output*_*Type*), hyphen and underscore (*Anti-adhesion_characteristics*), Pascal case (*RelationalConcept*), and combinations (*Small_cicada___Okinawa_*). Second, class labels with closely related meanings are not clearly distinguished. For instance, the differences between *Quality*, *Attribute*, and *Characteristics* are not specified. Next, class labels are inconsistently expressed both in singular and plural forms, as well as in lower- and uppercase, such as *Characteristics*, *fluid*, *elephant*, *Information-related_functions*, and *Mechanical_function*. Fourth, some labels are excessively long, such as *Power_generation_with_no_harm_to_the_surrounding_environment* or *Dark_brown_thorns_edge_giant_thin_blade_Beetle*, while others, like *Specific goal*, cover a wide range of topics without any specification. Some labels, like *Photoreceptor*, are too specific, while others, like *Application-dependent*, are vague and overly broad. Lastly, some classes with different URIs and different Japanese labels share the same English label. For instance, there are two classes with the English label *Bacteria*, one of which occurs as a highest class of the Organism Ontology (細菌), while the other is a subclass of *Animal* (バクテリア). Similarly, the label *Aquatic* is used both for a subclass of *Living_environment* (水生) and for a subclass of *Terrestrial* (水上). The distinction between these terms may be clear to Japanese speakers—the former likely refers to animals living in water, and the latter to animals that can live on the water’s surface—but the English labels do not reflect these nuances.

In addition to these inconsistencies, the OET violates other OBO Foundry Naming Conventions, such as using negation in the labels (*Non-poisonous*), catch-all terms (*UndefinedClass*) and parenthesis in labels like “構造色(?)”, whose English label is *Structural_color*, and カギツメヒゲブトコメツキ(仮称) and 昆虫ミメティクス(昆虫), whose English labels are *Key_claw_beard_but-click_beetle__tentative_name_* and *Insect_mimetics__insects_* respectively. “__[word]_” in the English label is equivalent to “(word)” in the Japanese label. Finally, for some classes, Japanese names were used as labels (e.g., *Kokuroshidemushi*, *Matsumomushi*, and *Miyako_Niinii*), which is not easily understandable for non-Japanese users.

**Clarity:** Language barrier and lack of documentation compel assessment to some extent, but the OET ontologies fail in this criterion due to arbitrary and vague class labels. For instance, it is not apparent that *Insect_mimetics* and *Goal* are under the same class. And in the subsumption hierarchy *Sensory hair* < *Part* < *Organism*, the class *Part* leads to confusion about how certain bodily entities should be classified and how they are related to *Organism* with an **is a** hierarchy, as it fuses taxonomy and partonomy. The same problem also occurs in *Adult_and_larva* < *Biological_attribute*. Moreover, there are vague class labels: *Other_attributes* (<*Physical_attributes*) or *UndefinedClass* (<*owl*:*Thing*) are ambiguous and too vague. *Recognition_of* is not complete, like *Application_dependent* and *Large*. Several classes are not mutually excluded and are vaguely labelled, as seen in the examples of *Cold_area* and *House_around* as living environments. Next, *Microorganism* is a subclass of *Animal* in Organism Ontology, yet microorganisms are not limited to animals, plants, or bacteria; they represent a wide variety of life forms, including other categories like fungi and protozoa. Thus, the overall evaluation of class labels suggests that the alleged subclasses represent a mere list of entities rather than an attempt to classify those entities systematically.

**Machine readability:** The ontologies are encoded in Hozo format, which is accessed via the API of the same name. From there, the OET can be exported in OWL, RDSF, and other machine-readable formats.

**Interoperability:** The OET ontologies are, in principle, interoperable with other semantic tools based on YAMATO. However, its inconsistent structure and ambiguous semantics prevent interoperability of the OET ontologies.

**Extensibility:** Its modular structure and its use of YAMATO as a top-level ontology would facilitate extensions of the OET ontologies, particularly its use together with other YAMATO-based ontologies. However, this is seriously challenged by the OET’s labelling problems and the problems with the taxonomic structure.

#### 4.4.5. The BID Ontology

Chen et al. [83] develop a knowledge graph for bio-inspired design intended as a structured framework for efficient knowledge retrieval, supporting reasoning capabilities and enabling designers to identify new applications for specific biological features. This knowledge graph is based on an ontology, which is used to build the knowledge graph and serves as a basis for extracting and categorising cross-domain entities from BID knowledge bases using large language models.

This BID Ontology contains five highest genera: *Source*, *Benefits*, *Application*, *Biological category*, and *Application categories*. Of these, *Source*, *Benefits*, and *Application* are said to be primary, as they capture the most crucial information within a BID case. *Biological category* mirrors the Linnean taxonomy, and *Application categories* comprises nine topical fields for patent categorisation to classify the source (i.e., the organism or biological concept generator) and the application.

The BID Ontology defines various relations among these categories or their entities, which can be distinguished according to their domain and range: (i) *Source* to *Benefits* (S-r-B) that connects a biological source to its associated benefits, (ii) *Benefits* to *Application* (B-r-A) that links the benefits derived from a biological source to their practical applications, (iii) *Source* to *Biological category* (S-r-C) that categorises the biological sources within a broader biological classification, and (iv) *Application* to *Application categories* (A-r-C) that connects specific applications to broader categories of applications.

*Source* contains specific biological entities, such as animals, plants, or ecosystems, that offer insights for design solutions. *Benefits* has six immediate subcategories, which capture attributes offered by the entities of *Source*: *Function*, *Structure*, *Shape*, *Color*, *Texture*, and *Imagery*, also referred to as “incentive perspectives”. These perspectives guide relationships that connect biological features with actionable design principles. The relations that link *Benefits* classes to *Source* entities are **has function**, **has structure**, **has shape**, **has color**, **has texture**, and **has imagery,** and that link *Benefits* classes to *Application* entities are **functional BID**, **structured BID**, **shaped BID**, **colorful BID**, **textured BID**, and **imaginative BID**. *Application* contains entities of the practical implementation of the insights gained from biological sources in innovation or design solutions in technology, architecture, engineering, or other fields.

*Biological category* has seven monohierarchically categorised immediate subclasses: *Kingdom*, *Phylum*, *Class*, *Order*, *Family*, *Genus*, and *Species*. This branch serves to organise biological entities of the *Source* class from kingdom to species level. The hierarchical classes and the entities of the *Source* class are related to each other by the **belongs to kingdom**, **belongs to phylum**, **belongs to class**, **belongs to order**, **belongs to family**, **belongs to genus**, and **belongs to species** relations.

*Application categories* is based on the Cooperative Patent Classification (CPC) system, a patent categorisation framework (https://www.cooperativepatentclassification.org, accessed on 3 January 2025). The CPC consists of nine main sections, which are further divided into subsections and contains approximately 250,000 classification entries, some of which may appear in multiple sections. The immediate subclasses of the *Application categories* are the nine main sections of the CPC: *Human necessities*, *Performing operations*/*Transporting*, *Chemistry*/*Metallurgy*, *Textiles*/*Paper*, *Fixed constructions*, *Mechanical engineering*/*Lighting*/*Heating*/*Weapons*/*Blasting*, *Physics*, *Electricity*, and *Emerging technology*, which are mutually exclusive (p. 12). Similar to how *Biological category* organises *Source* entities, *Application categories* organises *Application* entities: These categories group *Application* entities that can emerge from the identified entities of *Benefits*.

The entities under *Benefit*, *Source*, and *Application* were extracted by three distinct GPT-3 models that were fine-tuned with human-labelled data from 200 BID cases of AskNature by Chen et al. [84]. *Benefit* entities were categorised using a GPT-4 prompt that includes six incentive perspectives and their definitions as input data, instruction of the task, and few-shot task examples. Similarly, *Application* entities were categorised using a GPT-4 prompt that includes nine categories of *Application categories* and their definitions as input data and instruction for the task. Lastly, *Source* entities are categorised by querying the Catalogue of Life (https://www.catalogueoflife.org, accessed on 3 January 2025), an online biological taxonomy database that stores the names and corresponding taxonomical categories of *Biological category*.

**Availability:** The ontology is publicly unavailable. All information about the ontology given here is taken from [83].

**Documentation:** The only source for the ontology is [83]. The term definitions are provided for prompting. The domain and range restrictions of the relations are illustrated in the paper.

**Taxonomy inclusion:** The taxonomy of the BID Ontology is unorthodox. The classes *Biological category* and *Application categories*, as well as the incentive perspectives provide metadata to the entities of *Source*, *Application*, and *Benefit* respectively. Such data are annotated automatically either by prompts of GPT-4 or queries. Entities of *Source* and *Application* are not grouped into immediate subclasses; they are rather classified through the taxonomical structure of the *Biological category* and *Application categories*, respectively.

The immediate subclasses of *Application categories* are considered to be mutually exclusive in the BID Ontology [83]. However, the definitions in figure 6 of [83] suggest that some overlap between these classes is inevitable. For instance, it seems that an instance of *Mechanical engineering*/*Lighting*/*Heating*/*Weapons*/*Blasting* can also be an instance of *Chemistry*/*Metallurgy*, or *Physics*, or *Electricity*, or *Emerging technology*. It is even explicitly said that *Physics* “covers […] electricity” ([83], p. 14), but still, *Electricity* and *Physics* are treated as mutually disjoint classes.

**Logical consistency:** The BID Ontology was unavailable for automated checking, and the logical constraints of the relations involved were not documented. However, the taxonomy problems mentioned could lead to inconsistencies.

**Consistent labelling:** There is no singular–plural consistency of the labels. Although entities of *Source* and *Application* were automatically extracted, a normalisation could have been applied to preserve consistent labelling.

The subclass labels of the *Application categories* violate the OBO Foundry Naming Conventions. Above all, some labels are not understandable, long, and constructed with enumerations, such as in the label of the class *Human Necessities*, of the class *Textiles; Paper*, or of the class *Mechanical engineering; Lighting; Heating; Weapons; Blasting*. Even though white space is used to separate words, the labels begin with uppercase letters. There are labels in plural noun form, such as *Human Necessities* or *Fixed constructions*.

**Clarity:** The class labels under the *Application categories* are problematic. For instance, there can be overlapping categories, such as *Emerging technology* can overlap with any other categories depending on the type of technology. *Physics* is more general than *Mechanical engineering*/*Lighting*/*Heating*/*Weapons*/*Blasting*, which is overly specific; the labels reflect inconsistent granularity, so the labels that cover a wide range of topics lead to confusion. Though used in prompting, the definitions of the nine *Application categories* are not clearly presented for human comprehension.

The relations used are not sufficiently explained; for example, it seems strange that the **has function** relation is asserted between *Australian frogs* and *Harden quickly* or *Encourage healing*, as neither of them is, in fact, a function of these frogs (figure 4, [83]). Additionally, the statement “*butterflies* **has color** *high reflectivity*” (figure 7, [83]) is inadequate, as high reflectivity is not a colour. There is also a whole group of relations with “BID” in their labels whose semantics is quite opaque, like “structured BID” or “colorful BID”. Lastly, the relations from and to *Benefit* appear to be bidirectional. For instance, figure 2 in [83] suggests that the domain of the **has structure** relation is *Source*, whereas figure 7 in [83] provides an example where the range of the **has structure** relation is *Source*. This bidirectional nature of relations holds for all the relations from and to *Benefit*.

**Machine readability:** Storage of the knowledge graph and its testing in the case studies suggest that the entity relation triples of the BID Ontology are represented in a machine-readable format. No information about the specific storage format is given.

**Interoperability:** The BID Ontology seems to lack a standard language, which hinders its interoperability.

**Extensibility:** The modular structure of the ontology allows extensibility.

## 5. Discussion

Using the criteria introduced in Section 3.2, we evaluated nine terminological resources that have been developed for Biologically Inspired Design or biomimetics. The results are summarised in Table 5. This synopsis shows that six of nine resources are available. Seven terminological resources are documented for use or reuse; of these, only BMO is documented in a separate file as a guideline. Many resources are only documented in research papers, without full coverage of the definitions of classes and relations. These two observations alone hinder the reusability of the respective resources.

Having a taxonomic backbone is essential for (reuse as) an ontology. The lack of a coherent **is a** hierarchy hinders the fulfilment of the criteria of logical consistency, clarity, and interoperability. None of the terminological resources fulfils all desiderata in this respect. Many tools deal too light-handedly with the relations between the classes. For instance, the monohierarchical structures of, say, the Biomimicry Taxonomy, the E2B Thesaurus, or the BMO, are constructed without a set-theoretical foundation such that classes do not reflect an **is a** structure. As can be clearly seen from the example in the OET ontologies, the classes are at times presented as mere vocabulary collections without consideration of the implicit or explicit semantics of the ontology description language used.

Logical consistency evaluates the internal coherence of a terminological resource, which is essential for flawless formalisation. For this reason, it serves as a hidden prerequisite for use and reuse, particularly for clarity and interoperability. Of the two available machine-readable ontologies, the OET file yields inconsistencies when used with an automated reasoner. Although the BMO did not produce such results, the lack of domain and range restrictions of relations poses an obstacle to determining its logical consistency. None of the other resources are represented in a machine-readable format—directly or indirectly—so they were assessed manually. The most common error observed is the reoccurrence of a class at different levels, which is followed by the incorrect usage of the **is a** relation. A defined class, namely a logical combination of at least two classes, should not be positioned within a taxonomy.

We found that class labels are often unsystematic and incomprehensible. The most common error is the lack of uniformity in style. There are instances where labels appear in both singular and plural forms, employ various word separators, and exhibit inconsistent use of lower- and uppercase letters. In other instances, some labels are expressed in negative or disjunctive forms or by means of plural forms or abbreviations. In many cases, entity labels are not short, memorable, or understandable. Overall, only the SBF schema employs a consistent style. None of the resources, however, complied with the OBO Foundry Naming Conventions. In particular, the abundant use of verbs for functions is in conflict with the requirement that class labels should be referring to expressions, i.e., nouns or noun phrases. The absence of consistent labelling patterns hinders the fulfilment of the criteria of clarity and extensibility. This lack not only leads to ambiguities with respect to the intended meaning of class and property labels, as well as definitions, but also to incorrect classifications and the wrong usage of the **is a** relation.

Clarity is the second criterion that all terminological resources fail to satisfy. This criterion is crucial for use and reuse, especially for ensuring interoperability. The most significant issue with respect to clarity is that class labels and definitions often cause ambiguity. Many class labels are unclear and frequently do not correspond to the same categories of entities in the real world. Context-dependent class labels often fail to align with their intended meanings: The genus–differentia labelling style is often not maintained. As a result, definitions become either unintelligible or conflicting, and often **is a** overload is observed, i.e., the use of the subsumption relation in cases where other formal relations should be used in order to avoid polyhierarchies. The ambiguity of labels and definitions requires extra cognitive effort to grasp their meanings. Furthermore, in ontologies, the lack of constraints on the relations impairs clarity, as the absence of domain and range specifications leads to incorrect usage.

Only four of the resources are machine-readable; of these, only two are available, though not publicly. The existence of structured but not machine-readable resources shows that there is a requirement for an ontology aligning these terminological resources.

Interoperability and extensibility are crucial for reuse. Most resources are not built using standard formats or protocols, as they are designed primarily for human comprehension rather than as computational tools. The machine-readable resources, by definition, apply some level of standardisation, but an inconsistent taxonomical structure or ambiguous terms can hinder their interoperability. Using a standard top-level ontology or schema as a basis of a terminological resource enhances, but does not guarantee, interoperability of the resource. Interoperability also requires both semantic and syntactic alignment through shared annotations, as well as logical consistency and thorough documentation. Without these, achieving seamless interaction across terminological resources and other semantic tools remains a significant challenge that can be overcome by building a reference ontology for biomimetics.

Except for the Biomimicry Taxonomy, all the resources offer some degree of extensibility. This is often facilitated by a modular architecture and the use of standards like top-level ontologies or schemata. However, thorough documentation is essential for ensuring extensibility, which is provided only by the SAPPhIRE model and the UNO-BID; the latter is indeed the extension of the former.

In order to support the biomimetic development process, a reference ontology needs to represent the central categories of biomimetics. Following a standard analysis of the construction process in engineering, Drack et al. [85] suggest that the central concepts from this analysis (namely, function, working principle, and construction) can also be used to describe. It has been shown that this description fits prominent examples in biomimetics [86]. All of the terminological resources cover functions, either biological, technical, or both, but working principles are explicitly mentioned in the SBF schema, and they are also structured in the BMO and the Ontology for BID, but none of these terminological resources ontologically analyse them. Constructions can be represented as classes, such as the Taxonomy of Organisms of the OET categorises organisms; or as defined classes, such as the Biomimetic Taxonomy annotates biological systems; or through a model, such as a technical system can be represented by the SAPPhIRE model. A future domain ontology of biomimetics should contain representational units for all of these entities. In previous work, though, we have shown that biomimetics does not require a unified account of biological and technical functions [87]. Nevertheless, a solid classification of process types will be crucial for a biomimetic ontology, and existing resources like the Process Specification Language (PSL) and the Biological Process branch of the Gene Ontology should be checked for potential reuse within a reference ontology for biomimetics. Ideally, the reference ontology integrates as many existing terminological resources as possible by reusing them or by providing bridges to them. This applies especially to tools like the Biomimicry Taxonomy, which is already used to annotate the contents of the rich AskNature database.

## 6. Conclusions

Biomimetic knowledge deserves to be structured according to its own categories. Our analysis of existing terminological resources for structuring biomimetic knowledge shows that no existing resource can adequately represent biomimetic knowledge due to its content, scope, or structure. In particular, no adequate domain ontology suitable to support the biomimetic development process exists. Consequently, an adequate reference ontology for the domain of biomimetics that has a general functional theory and expounds all relevant constituents of biomimetic knowledge remains a desideratum. A reference ontology can solve the interoperability of various databases and helps to avoid data silos. It will be able to align differently labelled and/or categorised functions, working principles, and constructions, thereby also bridging the differences between biology and engineering.

Sometimes, the hope is aired that state-of-the-art artificial intelligence, in particular large language models (LLMs) and other generative AI models, reduces the need for ontologies in effectively integrating, managing and using both biological and technological knowledge, as these models, after all, can often infer patterns and relationships in data without requiring formal, predefined structures. However, LLMs are connectionist systems based on neural network models, i.e., statistical models trained on masses of data. For this reason, they are normally very bad in logical reasoning, including conceptual logic and the recognition of synonyms. In contrast, ontologies are symbolic systems designed to represent knowledge in a formally structured way, making them particularly capable of supporting logical reasoning.

These two paradigms thus operate on different principles, and they serve different roles in knowledge representation. First, ontologies ensure machine readability by explicitly structuring data, classifying them, and determining relations between the classes and the relation properties used. LLMs, on the other hand, do not structure data but rather statistically organise them. In one sense, ontologies mirror scientific categorisations of the real world, while LLMs reflect the data in which they are trained. Thus, the reliability of the latter models is questionable and not as reliable as required.

Second, ontologies include axioms describing the relations between entities, the properties of these relations, and other formal constraints. Hence, detecting inconsistencies in a logic-based model is straightforward but, most of the time, unavailable in a statistical model. Indeed, a significant limitation of LLMs lies in their inability to determine upper categories or properly define relation properties. For instance, they struggle to differentiate between different kinds of relation [88], which is a cornerstone of ontology design.

For these reasons, the critical importance of ontologies remains widely recognised as today’s AI models still lack explainability, interdisciplinary communication, reliability, and logical consistency, where ontologies excel.

AI tools could, and should, be used in combination with ontologies to elevate the shortcomings mentioned. The integration of ontologies with generative AI models creates a fruitful synergy that leverages the potential of the latter models. Ontologies can complement LLMs, particularly in taming their outputs or enhancing their capabilities during training or fine-tuning. They ensure the represented knowledge is explicit, logically consistent, reusable, and structured using a shared vocabulary and definitions. These features ensure greater precision and contextual relevance and are thus crucial for interdisciplinary domains like biomimetics. LLMs on their own normally lack them. While LLMs can assist in generating ontologies, much like they can write a poem or compose text, the ontologies they produce are not inherently reliable. Their internal consistency and logical coherence must be rigorously verified. This highlights the need for caution and expert validation when incorporating LLM-generated elements into ontology development, as well as the enduring need for reliable reference ontologies.

Future work should develop such a resource for the domain of biomimetics. The evaluation made it drastically clear that long-term availability is crucial for any sensible computational tool to be developed. For this reason, it is desirable that a future domain ontology for biomimetics is provided through reliable repositories, ideally under an appropriate Creative Commons license and adhering to the FAIR principles [89]. This requires, among other things, sufficient documentation of the ontology, both within the ontology file and in separate text documents, as well as appropriate standards for storage, versioning, community support, and modular architecture. Compliance with the Open Biological Ontology (OBO) Foundry Principles (https://obofoundry.org/principles/fp-000-summary.html, accessed on 3 January 2025) would allow a domain ontology for biomimetics to integrate with a large number of already existing OBO Foundry ontologies. The good practice rules of the OBO Foundry include their naming conventions and also advise using a top-level ontology like Basic Formal Ontology [90]. It should be taken care that the intended semantics of the top-level categories are adhered to in order to avoid modelling errors. Our survey showed that presently, no such terminological resource exists. This makes the integration of resources or data annotated by these resources presently challenging.

## Figures and Tables

**Table 1:** The tools evaluated and their characteristics.

Tools	Type	Status	Number of Classes	Number of Relations	Taxonomy Included	Machine Readable
The Biomimicry Taxonomy	Taxonomy	Actively used, open source	162	1	No	No
The Engineering-to-Biology Thesaurus (E2B)	Thesaurus	Actively used, open source	48 function classes 38 flow classes	1	No	No
The SBF Schema	Schema	Actively used, open source	10	1	No	Not currently but formally specified
The SAPPhIRE Model	Schema	Actively used, open source	7	3	No	Not currently but formally specified
The Unified Ontology for BID (UNO-BID)	Ontology	Unavailable	30	2	No	Yes (format unknown)
The Ontology for BID	Ontology	Unavailable	Over 92	10	No	Yes (OWL)
The BioMimetics Ontology (BMO)	Ontology	Ongoing project, available upon request	Over 3000	Over 50	No	Yes (OWL)
The Ontology-Enhanced Thesaurus (OET) *	Ontology	Available upon request	Over 2000	80	No	Yes (native format Hozo; can be exported in OWL)
The BID Ontology	Ontology	Unavailable	Over 27	21	No	Yes (format unknown)

OWL: Web Ontology Language; * Numbers are based on the Protégé metrics for the OWL version.

**Table 2:** Evaluation criteria for biomimetic terminological resources.

Criterion	Measurement Method
**Infrastructure**	
**Availability** verifies that the resource is accessible and/or usable when needed.	Check whether the resource is downloadable and/or its structure can be obtained from the literature.
**Documentation** includes providing clear and comprehensive instructions for supporting users, facilitating understanding and proper utilisation.	Investigate any explanatory resources, such as GitHub repositories, webpages, or research papers.
**Content Representation**	
**Taxonomy inclusion** evaluates whether there is an **is a** hierarchy culminating in mutually exclusive terms.	Conduct through semantic analysis by manually checking if each node follows the **is a** condition from top-level terms.
**Logical consistency** guarantees that there are no internal inconsistencies in the resources.	If possible, use automated reasoner to check for consistency, or else check manually.
**Consistent labelling** checks whether coherent conventions are adhered to in terms and definitions.	Evaluate terms and definitions manually.
**Clarity** evaluates how clearly and effectively the terminological resource communicates the intended meaning of the term.	Check that the definitions of terms, relations, and annotations are clear, unambiguous, and understandable to humans.
**Reusability**	
**Machine-readability** ensures that the terminological resource is formalised and represented in the machine to enable utilisation of its content by automated systems and for computational processes.	Check whether the resource is represented in a machine-readable format and whether it complies with the syntactical rules of the chosen formats.
**Interoperability** evaluates how well a terminological resource is prepared to be used together with other semantic tools to exchange data within and across domains.	Check whether the resource follows standard data formats or protocols.
**Extensibility** ensures that a terminological resource can accommodate extensions due to increasing domain knowledge and changing user requirements.	Check whether guidelines or best practices are provided for making extensions and whether the resource is built modularly.

**Table 3:** Examples from the Biomimicry Taxonomy. Items under the headings of “Group”, “Subgroup”, and “Function” are taken from the taxonomy; items under “Function Term” have been scraped from the AskNature search page (https://asknature.org, accessed on 23 December 2023).

Group	Subgroup	Function	Function Term
*Process information*	*Compute*		Compute
*Move or stay put*	*Move*	*In*/*on solids*	Move in/on solids
*Protect from physical harm*	*Protect from living threats*	*Microbes*	Protect from microbes
*Perform*	*Process signals*	*Respond to signals*	Respond to signals

**Table 4:** A selection from the E2B Thesaurus. The degree of specification increases from the left to the right. Sourced from [44], with definitions from [47]. Each iterative division represents an increasing degree of specification in the function or flow.

Class	Secondary	Tertiary	Biological Function Correspondents
**Function Terms**			
Branch To cause a flow (material, energy, signal) to no longer be joined or mixed.	Separate To isolate a flow (material, energy, signal) into distinct components.		Bleaching, meiosis, abscission, mitosis, segment, *electrophoresis*, *dialysis*, denature, free, detach, release
		Divide To separate a flow.	*Divide*, prophase, metaphase, *anaphase*, cleave, cytokinesis
		Remove To take away a part of a flow from its prefixed place.	Deoxygenate, filtrate, liberate, expulsion, evacuate
	Distribute To cause a flow (material, energy, signal) to break up.		*Circulate*, *diffusion*, *exchange*, disperse, scatter, spread, spray
Convert To change from one form of a flow (material, energy, signal) to another.	Convert		Polymerize, […] *degrade*, *develop*, *mutate*, *photosynthesize*
**Material Flow Terms**			
Material	Solid Any object with mass having a definite, firm shape.	Particulate Substance containing minute separate particles	Cytokinin, […] *RNA*, tRNA, mRNA, *DNA*, […], enzyme, […]
		Composite Solid material composed of two or more substances having different physical characteristics and in which each substance retains its identity while contributing desirable properties to the whole unit.	*Enzyme*, […], *DNA*, *RNA*, cytoplasm, *organ*, *tissue*
	Human All or part of a person who crosses the device boundary.		Being, *body*
**Energy Flow Terms**			
Energy	Human Work performed by a person on a device.		
	Electrical Work resulting from the flow of electrons from a negative to a positive source.		Electron, potential, feedback, charge, field

**Table 5:** Overview of the evaluation of the terminological resources. ++: The criterion is fulfilled. +−: The criterion is partially applicable. −−: This criterion is not fulfilled. ?: The criterion cannot be decided. Detailed description and justification in Section 4.

	Criteria								
	Infrastructure		Content Representation				Reusability		
Terminological Resources	Availability	Documentation	Taxonomy Inclusion	Logical Consistency	Consistent Labelling	Clarity	Machine Readability	Interoperability	Extensibility
The Biomimicry Taxonomy	++	+−	−−	−−	−−	−−	−−	−−	−−
Engineering-to-Biology Thesaurus	++	++	−−	−−	+−	−−	−−	+−	+−
The SBF Schema	++	+−	−−	++	+−	−−	+−	+−	++
The SAPPhIRE Model	++	++	−−	++	−−	−−	+−	+−	++
The UNified Ontology for BID	−−	++	−−	++	−−	−−	++	+−	++
The Ontology for BID	−−	−−	−−	+−	−−	−−	++	+−	++
The BioMimetics Ontology	+−	+−	−−	++	−−	−−	++	+−	++
The Ontology-Enhanced Thesaurus	+−	+−	−−	−−	−−	−−	++	+−	+−
The BID Ontology	−−	−−	−−	?	−−	−−	++	−−	++

## Data Availability

The original contributions presented in the study are included in the article. Further inquiries can be directed to the corresponding author.

## References

[1] (2012). Bionik. Konzeption und Strategie. Abgrenzung Zwischen Bionischen und Konventionellen Verfahren/Produkten (Biomimetics. Conception and Strategy. Differences Between Biomimetic and Conventional Methods/Products).

[2] Fensel D. (2004). Ontologies.

[3] Leonelli S. (2016). Data-Centric Biology: A Philosophical Study.

[4] Schulz S., Jansen L. (2013). Formal Ontologies in Biomedical Knowledge Representation. Yearb. Med. Inform..

[5] Smith B., Ashburner M., Rosse C., Bard J., Bug W., Ceusters W., Goldberg L.J., Eilbeck K., Ireland A., Mungall C.J. (2007). The OBO Foundry: Coordinated evolution of ontologies to support biomedical data integration. Nat. Biotechnol..

[6] Ashburner M., Ball C.A., Blake J.A., Botstein D., Butler H., Cherry J.M., Davis A.P., Dolinski K., Dwight S.S., Eppig J.T. (2000). Gene Ontology: Tool for the unification of biology. Nat. Genet..

[7] Fayemi P.E., Wanieck K., Zollfrank C., Maranzana N., Aoussat A. (2017). Biomimetics: Process, tools and practice. Bioinspir. Biomim..

[8] (2023). Bionik. Bionische Entwicklungsmethodik. Produkte und Verfahren (Biomimetics. Biomimetic Design Methodology. Products and Processes).

[9] Wanieck K., Fayemi P.-E., Maranzana N., Zollfrank C., Jacobs S. (2017). Biomimetics and its tools. Bioinspir. Biomim. Nanobiomater..

[10] McGuinness D.L., Fensel D., Hendler J.A., Lieberman H., Wahlster W. (2003). Ontologies Come of Age. Spinning the Semantic Web.

[11] Kundisch D., Muntermann J., Oberländer A.M., Rau D., Röglinger M., Schoormann T., Szopinski D. (2022). An Update for Taxonomy Designers: Methodological Guidance from Information Systems Research. Bus. Inf. Syst. Eng..

[12] (2011). Information and Documentation—Thesauri and Interoperability with Other Vocabularies–Part 1: Thesauri for Information Retrieval.

[13] Aitchison J., Bawden D., Gilchrist A. (2000). Thesaurus Construction and Use.

[14] Kless D., Milton S., Sánchez-Alonso S., Athanasiadis I.N. (2010). Towards Quality Measures for Evaluating Thesauri. Metadata and Semantic Research.

[15] Owens L.A., Cochrane P.A. (2004). Thesaurus Evaluation. Cat. Classif. Q..

[16] Shchitov I., Lagutina K., Lagutina N., Paramonov I., Vasilyev A. A survey on thesauri application in automatic natural language processing. Proceedings of the 21st Conference of Open Innovations Association (FRUCT).

[17] Spyns P., Meersman R., Jarrar M. (2002). Data modelling versus ontology engineering. SIGMOD Rec..

[18] Ford T.C., Colombi J.M., Graham S.R., Jacques D.R. A Survey on Interoperability Measurement. Proceedings of the Twelfth International Command and Control Research and Technology Symposium (12th ICCRTS).

[19] Gannon T., Madnick S., Moulton A., Siegel M., Sabbouh M., Zhu H. (2009). Framework for the Analysis of the Adaptability, Extensibility, and Scalability of Semantic Information Integration and the Context Mediation Approach. Proceedings of the 2009 42nd Hawaii International Conference on System Sciences.

[20] Liu L., Li W., Aljohani N.R., Lytras M.D., Hassan S.-U., Nawaz R. (2020). A framework to evaluate the interoperability of information systems—Measuring the maturity of the business process alignment. Int. J. Inf. Manag..

[21] Szopinski D., Schoormann T., Kundisch D. (2019). Because Your Taxonomy is Worth It: Towards a Framework for Taxonomy Evaluation. Proceedings of the 27th European Conference on Information Systems (ECIS).

[22] Kaplan A., Kühn T., Hahner S., Benkler N., Keim J., Fuchß D., Corallo S., Heinrich R. Introducing an Evaluation Method for Taxonomies. Proceedings of the EASE 2022: The International Conference on Evaluation and Assessment in Software Engineering.

[23] Stella G., Clarke D. (2011). ISO 25964: A standard in support of KOS interoperability. Facets of Knowledge Organization, Proceedings of the ISKO UK Second Biennial Conference, London, UK, 4–5 July 2011.

[24] Gangemi A., Catenacci C., Ciaramita M., Lehmann J. A theoretical framework for ontology evaluation and validation. Proceedings of the Semantic Web Applications and Perspectives.

[25] Brank J., Grobelnik M., Mladenic D. A survey of ontology evaluation techniques. Proceedings of the Data Mining and Data Warehouses (SiKDD 2005).

[26] Schober D., Smith B., Lewis S.E., Kusnierczyk W., Lomax J., Mungall C., Taylor C.F., Rocca-Serra P., Sansone S.-A. (2009). Survey-based naming conventions for use in OBO Foundry ontology development. BMC Bioinform..

[27] Vrandečić D., Staab S., Studer R. (2009). Ontology Evaluation. Handbook on Ontologies.

[28] Iqbal R., Murad M.A.A., Mustapha A., Sharef N.M. (2013). An Analysis of Ontology Engineering Methodologies: A Literature Review. Res. J. Appl. Sci. Eng. Technol..

[29] Raad J., Cruz C. (2015). A Survey on Ontology Evaluation Methods. IC3K 2015, Proceedings of the 7th International Joint Conference on Knowledge Discovery, Knowledge Engineering and Knowledge Management, Lisbon, Portugal, 12–14 November 2015.

[30] Ashraf J., Hussain O.K., Hussain F.K., Chang E.J. (2018). Measuring and Analysing the Use of Ontologies: A Semantic Framework for Measuring Ontology Usage. Studies in Computational Intelligence.

[31] Smith M., Horrocks I., Krötzsch M., Glimm B. (2012). OWL 2 Web Ontology Language Conformance.

[32] Vandevenne D., Pieters T., Duflou J.R. (2016). Enhancing novelty with knowledge-based support for Biologically-Inspired Design. Des. Stud..

[33] Deldin J.-M., Schuknecht M., Goel A.K., McAdams D.A., Stone R.B. (2014). The AskNature Database: Enabling Solutions in Biomimetic Design. Biologically Inspired Design.

[34] Taxonomy Explainer. https://asknature.org/wp-content/uploads/2021/06/Taxonomy_Explainer_2021.pdf.

[35] (2020). Biomimetics—Ontology Enhanced Thesaurus (OET) for Biomimetics.

[36] Vincent J.F.V. (2012). Structural Biomaterials.

[37] Drack M., Limpinsel M., de Bruyn G., Nebelsick J.H., Betz O. Glossary of Architectural and Constructional Biomimetics.

[38] Shu L.H., Cheong H., Goel A.K., McAdams D.A., Stone R.B. (2014). A Natural Language Approach to Biomimetic Design. Biologically Inspired Design.

[39] Cheong H., Chiu I., Shu L.H., Stone R.B., McAdams D.A. (2011). Biologically Meaningful Keywords for Functional Terms of the Functional Basis. J. Mech. Des..

[40] Wanieck K. (2019). In Bionik für Technische Produkte und Innovation: Ein Überblick für die Praxis (Essentials).

[41] Weidner B.V., Nagel J., Weber H.-J. (2018). Facilitation method for the translation of biological systems to technical design solutions. Int. J. Des. Creat. Innov..

[42] Kozaki K., Mizoguchi R., Supnithi T., Yamaguchi T., Pan J.Z., Wuwongse V., Buranarach M. (2015). A Keyword Exploration for Retrieval from Biomimetics Databases. Semantic Technology.

[43] Mizoguchi R., Kozaki K. (2022). Ontology-Enhanced Thesaurus for Promoting Biomimetics Research. Biomimetics.

[44] Nagel J.K., Stone R.B., McAdams D.A. (2010). An Engineering-to-Biology Thesaurus for Engineering Design. 2010 ASME International Design Engineering Technical Conferences and Computers and Information in Engineering Conference, Proceedings of the Volume 5: 22nd International Conference on Design Theory and Methodology, Special Conference on Mechanical Vibration and Noise, 15–18 August 2010, Montreal, QC, Canada.

[45] Stroble J.K., Stone R.B., McAdams D.A., Watkins S.E. (2009). An engineering-to-biology thesaurus to promote better collaboration, creativity and discovery. Proceedings of the 19th CIRP Design Conference–Competitive Design.

[46] Pahl G., Beitz W., Feldhusen J., Grote K.-H. (2007). Engineering Design.

[47] Hirtz J., Stone R.B., McAdams D.A., Szykman S., Wood K.L. (2002). A functional basis for engineering design: Reconciling and evolving previous efforts. Res. Eng. Design.

[48] Nagel J.K., Goel A.K., McAdams D.A., Stone R.B. (2014). A Thesaurus for Bioinspired Engineering Design. Biologically Inspired Design.

[49] Nagel J.K. Engineering to Biology Thesaurus. https://web.archive.org/web/20220414114402/https:/jacquelynnagel.com/wp-content/uploads/2020/10/E2BThesaurus.pdf.

[50] Nagel J.K. Engineering to Biology Thesaurus Version 2. https://web.archive.org/web/20211025094939/https://jacquelynnagel.com/wp-content/uploads/2020/10/E2BThesaurusv2.pdf.

[51] Nagel J.K. Bio-Inspired Design Resources. https://web.archive.org/web/20240712153507/https:/jacquelynnagel.com/bid-resources/.

[52] Garbacz P. (2006). Towards a standard taxonomy of artifact functions. Appl. Ontol..

[53] McInerney S.J., Khakipoor B., Garner A.M., Houette T., Unsworth C.K., Rupp A., Weiner N., Vincent J.F.V., Nagel J.K.S., Niewiarowski P.H. (2018). E2BMO: Facilitating User Interaction with a BioMimetic Ontology via Semantic Translation and Interface Design. Designs.

[54] Design & Intelligence Laboratory DANE 2.0 Users Guide. https://web.archive.org/web/20210704202220/http:/dilab.cc.gatech.edu/dane/files/DANE%202%20Users%20Guide.pdf.

[55] Chandrasekaran B. (1994). Functional representation: A brief historical perspective. Appl. Artif. Intell..

[56] Vattam S., Wiltgen B., Helms M., Goel A.K., Yen J., Taura T., Nagai Y. (2011). DANE: Fostering Creativity in and through Biologically Inspired Design. Design Creativity 2010.

[57] Design & Intelligence Laboratory DANE: Design Analogy to Nature Engine. https://web.archive.org/web/20230929200509/http:/dilab.cc.gatech.edu/dane/.

[58] Goel A.K., Rugaber S., Vattam S. (2009). Structure, behavior, and function of complex systems: The structure, behavior, and function modeling language. Artif. Intell. Eng. Des. Anal. Manuf. (AI EDAM).

[59] Petit-Bois R., Jacob J., Rugaber S., Goel A. Towards a Virtual Librarian for Biologically Inspired Design—Knowledge-Based Methods for Document Understanding. Proceedings of the SDU@ AAAI 2021.

[60] Goel A., Hagopian K., Zhang S., Rugaber S., Gero J.S. (2022). Towards a Virtual Librarian for Biologically Inspired Design. Design Computing and Cognition’20.

[61] Vattam S.S., Goel A.K., Rugaber S., Hmelo-Silver C.E., Jordan R., Gray S., Sinha S. (2011). Understanding Complex Natural Systems by Articulating Structure-Behavior-Function Models. J. Educ. Technol. Soc..

[62] Rugaber S., Bhati S., Goswami V., Spiliopoulou E., Azad S., Koushik S., Kulkarni R., Kumble M., Sarathy S., Goel A., Goel A., Díaz-Agudo M.B., Roth-Berghofer T. (2016). Knowledge Extraction and Annotation for Cross-Domain Textual Case-Based Reasoning in Biologically Inspired Design. Case-Based Reasoning Research and Development.

[63] Borgo S., Carrara M., Garbacz P. (2015). A formalization of Ashok Goel’s SBF concept of function. Proceedings of the 1st Workshop on Artificial Intelligence and Design (AIDE 2015).

[64] Jia L., Peng Q., Tan R., Zhu X. (2019). Analogical stimuli retrieval approach based on R-SBF ontology model. J. Eng. Des..

[65] Chakrabarti A., Sarkar P., Leelavathamma B., Nataraju B.S. (2005). A functional representation for aiding biomimetic and artificial inspiration of new ideas. Artif. Intell. Eng. Des. Anal. Manuf. (AI EDAM).

[66] Baldussu A., Cascini G., Rosa F., Rovida E. Causal models for bio-inspired design: A comparison. Proceedings of the DS 70: Proceedings of DESIGN 2012, the 12th International Design Conference.

[67] Bhattacharya K., Bhatt A.N., Ranjan B.S.C., Keshwani S., Srinivasan V., Chakrabarti A., Gero J.S. (2023). Extracting Information for Creating SAPPhIRE Model of Causality from Natural Language Descriptions. Design Computing and Cognition’22.

[68] Fu K., Moreno D., Yang M., Wood K.L. (2014). Bio-Inspired Design: An Overview Investigating Open Questions From the Broader Field of Design-by-Analogy. J. Mech. Des..

[69] Siddharth L., Chakrabarti A. (2018). Evaluating the impact of Idea-Inspire 4.0 on analogical transfer of concepts. Artif. Intell. Eng. Des. Anal. Manuf. (AI EDAM).

[70] Venkataraman S., Chakrabarti A. Sapphire–an approach to analysis and synthesis. Proceedings of the DS 58-2: Proceedings of ICED 09.

[71] Bhattacharya K., Chakrabarti A., Chakrabarti A., Singh V. (2023). Application of SAPPhIRE Model of Causality in the Design of Product-Service Systems. Design in the Era of Industry 4.0, Volume 3.

[72] Rosa F., Cascini G., Baldussu A. (2015). UNO-BID: Unified ontology for causal-function modeling in biologically inspired design. Int. J. Des. Creat. Innov..

[73] Yim S., Wilson J.O., Rosen D.W. (2008). Development of an Ontology for Bio-Inspired Design using Description Logics. Proceedings of the International Conference on Product Lifecycle Management.

[74] Wilson J., Chang P., Yim S., Rosen D.W. (2009). Developing a Bio-Inspired Design Repository Using Ontologies. Proceedings of the International Design Engineering Technical Conferences and Computers and Information in Engineering Conference.

[75] Vincent J.F.V., Goel A.K., McAdams D.A., Stone R.B. (2014). An Ontology of Biomimetics. Biologically Inspired Design.

[76] Vincent J.F.V. (2017). The trade-off: A central concept for biomimetics. Bioinspir. Biomim. Nanobiomater..

[77] Vincent J. (2023). Biomimetics with Trade-Offs. Biomimetics.

[78] Vincent J.F.V., Bogatyreva O.A., Bogatyrev N.R., Bowyer A., Pahl A.-K. (2006). Biomimetics: Its practice and theory. J. R. Soc. Interface..

[79] Vincent J., Cavallucci D., Cavallucci D., De Guio R., Koziołek S. (2018). Development of an Ontology of Biomimetics Based on Altshuller’s Matrix. Automated Invention for Smart Industries.

[80] Mungall C.J., Torniai C., Gkoutos G.V., Lewis S.E., Haendel M.A. (2012). Uberon, an integrative multi-species anatomy ontology. Genome. Biol..

[81] Chang A., Jeske L., Ulbrich S., Hofmann J., Koblitz J., Schomburg I., Neumann-Schaal M., Jahn D., Schomburg D. (2021). BRENDA, the ELIXIR core data resource in 2021: New developments and updates. Nucleic Acids Res..

[82] Mizoguchi R., Borgo S. (2022). YAMATO: Yet-another more advanced top-level ontology. Appl. Ontol. (AO).

[83] Chen L., Cai Z., Jiang Z., Sun L., Childs P., Zuo H. (2024). A knowledge graph-based bio-inspired design approach for knowledge retrieval and reasoning. J. Eng. Des..

[84] Chen L., Cai Z., Jiang Z., Long Q., Sun L., Childs P., Zuo H. (2023). A Knowledge-Based Ideation Approach for Bio-Inspired Design. Proc. Des. Soc..

[85] Drack M., Limpinsel M., De Bruyn G., Nebelsick J.H., Betz O. (2018). Towards a theoretical clarification of biomimetics using conceptual tools from engineering design. Bioinspir. Biomim..

[86] Drack M., Jansen L., Meder F., Hunt A., Margheri L., Mura A., Mazzolai B. (2023). Biomimetics Analyzed: Examples from an Epistemological and Ontological Perspective. Biomimetic and Biohybrid Systems.

[87] Yargan D., Jansen L. Does Biomimetics Require a Unified Account of Function?. Proceedings of the Joint Ontology Workshops (JOWO)—Episode X: The Tukker Zomer of Ontology, and Satellite Events Co-Located with the 14th International Conference on Formal Ontology in Information Systems.

[88] Berglund L., Tong M., Kaufmann M., Balesni M., Stickland A.C., Korbak T., Evans O. (2024). The Reversal Curse: LLMs trained on “A is B” fail to learn “B is A”. arXiv.

[89] Wilkinson M.D., Dumontier M., Aalbersberg I.J., Appleton G., Axton M., Baak A., Blomberg N., Boiten J.-W., Da Silva Santos L.B., Bourne P.E. (2016). The FAIR Guiding Principles for scientific data management and stewardship. Sci. Data.

[90] Arp R., Smith B., Spear A.D. (2015). Building Ontologies with Basic Formal Ontology.

